# A metabolic switch regulates the transition between growth and diapause in *C. elegans*

**DOI:** 10.1186/s12915-020-0760-3

**Published:** 2020-03-18

**Authors:** Sider Penkov, Bharath Kumar Raghuraman, Cihan Erkut, Jana Oertel, Roberta Galli, Eduardo Jacobo Miranda Ackerman, Daniela Vorkel, Jean-Marc Verbavatz, Edmund Koch, Karim Fahmy, Andrej Shevchenko, Teymuras V. Kurzchalia

**Affiliations:** 1 Max Planck Institute of Molecular Cell Biology and Genetics, Dresden, Germany; 2 Paul Langerhans Institute Dresden of the Helmholtz Zentrum München at the University Hospital and Faculty of Medicine Carl Gustav Carus of TU Dresden, Dresden, Germany; 3 Institute for Clinical Chemistry and Laboratory Medicine, University Clinic and Medical Faculty, TU Dresden, Dresden, Germany; 4 Present address: German Cancer Research Center (DKFZ), Heidelberg, Germany; 5 Institute of Resource Ecology at the Helmholtz-Zentrum Dresden-Rossendorf, Dresden, Germany; 6 Faculty of Medicine Carl Gustav Carus, Department of Anesthesiology and Intensive Care Medicine, Clinical Sensoring and Monitoring, TU Dresden, Dresden, Germany; 7 Institut Jacques Monod, Université de Paris/CNRS, Paris, France

## Abstract

**Background:**

Metabolic activity alternates between high and low states during different stages of an organism’s life cycle. During the transition from growth to quiescence, a major metabolic shift often occurs from oxidative phosphorylation to glycolysis and gluconeogenesis. We use the entry of *Caenorhabditis elegans* into the dauer larval stage, a developmentally arrested stage formed in response to harsh environmental conditions, as a model to study the global metabolic changes and underlying molecular mechanisms associated with growth to quiescence transition.

**Results:**

Here, we show that the metabolic switch involves the concerted activity of several regulatory pathways. Whereas the steroid hormone receptor DAF-12 controls dauer morphogenesis, the insulin pathway maintains low energy expenditure through DAF-16/FoxO, which also requires AAK-2/AMPKα. DAF-12 and AAK-2 separately promote a shift in the molar ratios between competing enzymes at two key branch points within the central carbon metabolic pathway diverting carbon atoms from the TCA cycle and directing them to gluconeogenesis. When both AAK-2 and DAF-12 are suppressed, the TCA cycle is active and the developmental arrest is bypassed.

**Conclusions:**

The metabolic status of each developmental stage is defined by stoichiometric ratios within the constellation of metabolic enzymes driving metabolic flux and controls the transition between growth and quiescence.

## Background

Throughout their life cycle, organisms alternate between states of high and low metabolic activity. In some cases, not only the intensity but the whole mode of metabolism changes, for instance, during the transition from growth to quiescence. Usually, growing organisms have highly active mitochondria and intensive oxidative phosphorylation (OXPHOS), whereas, during metabolic quiescence, they shift to glycolysis and associated gluconeogenesis [1, 2]. Most early embryos use glycolytic metabolism and only change to OXPHOS during later phases of development [3]. This metabolic shift is also observed during the differentiation of neurons and stem cells [4, 5]. The opposite change, from OXPHOS to aerobic glycolysis, is seen in cancer cells exhibiting “Warburg” metabolism [6]. Despite their importance, however, we still have a limited understanding of the mechanisms controlling these global metabolic transitions.

The entry of the nematode *Caenorhabditis elegans* into diapause is an excellent model in which to study these metabolic transitions. In response to harsh environmental conditions, *C. elegans* interrupts its reproductive life cycle, stops growing, and forms a specialized, developmentally arrested third larval stage called a dauer (enduring) larva [7]. The body of dauer larvae is morphologically adapted to harsh conditions. Its diameter is reduced, its body coated with a tight cuticle, and its pharynx sealed [7]. Most importantly, the metabolism of dauers differs substantially from that of the reproductive L3 larvae. Since they do not feed, they rely on stored energy reserves [7]. To restrict the depletion of these reserves, dauer larvae enter a hypometabolic mode via a dramatic rearrangement of anabolic and catabolic pathways [2, 8–13]. In this “standby” mode, energy consumption, heat production, aerobic respiration, and TCA cycle activity are significantly reduced. The production of cofactors required for anabolic reactions such as NADPH is also minimized [14]. Besides, the glyoxylate shunt and gluconeogenesis are used to generate carbohydrates from reserve lipids [2, 8–13]. In this state, dauers can survive for months without nutrition.

**Fig. 1:**
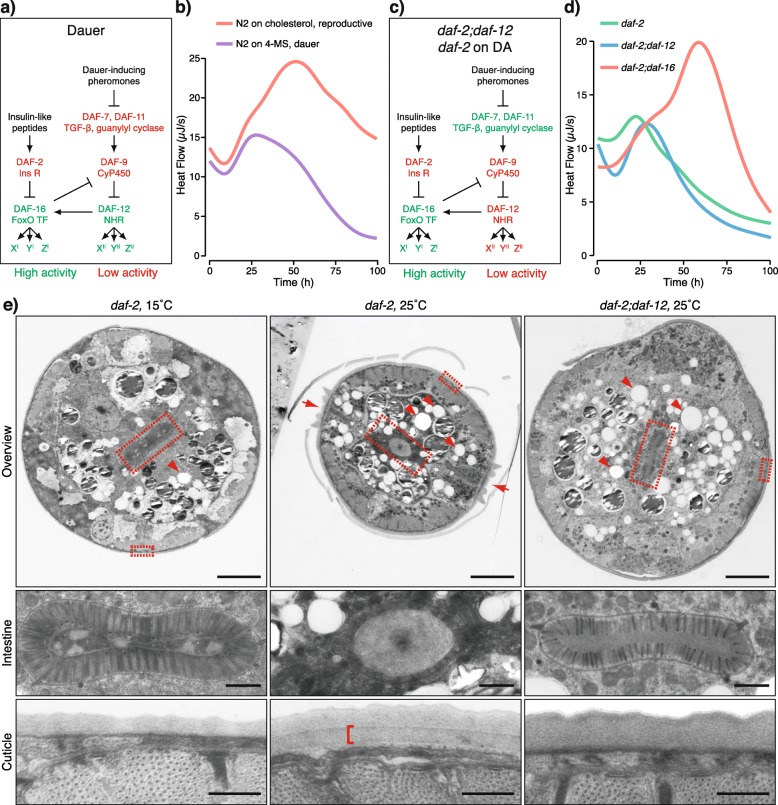
DAF-16 mediates the switch to low metabolic rate in dauer formation but does not directly induce dauer morphogenesis. **a** Signaling pathways in dauer formation. In the absence of insulin-like peptides, DAF-2 is suppressed, leading to the activation of DAF-16. Dauer-inducing pheromones lower the production of DA (dafachronic acid) by DAF-9, resulting in activated DAF-12. DAF-16 stimulates DAF-12 by inhibiting DAF-9. DAF-12 also activates DAF-16. DAF-16 and DAF-12 control subsets of genes X′Y′Z′ and X″Y″Z″, respectively. **b** Continuous measurement of the heat flow by wild-type (N2) worms undergoing reproductive growth on cholesterol or dauer formation on 4-MS (4-methylated sterol). Median heat flow of biological replicates from one experiment, smoothed using generalized additive models. Additional file 1: Fig. S1a displays biological replicates from various experiments. **c** Formation of L3 arrested larvae of *daf-2;daf-12* or DA-fed *daf-2* at the restrictive temperature. Inhibition of DAF-2 activates DAF-16. DAF-16 inhibits DAF-9, but this effect is neutralized by *daf-12* mutations or treatment with exogenous DA. **d** Heat flow of *daf-2*, *daf-2;daf-12*, and *daf-2;daf-16* grown at 25 °C. Median heat flow of biological replicates from one experiment, smoothed using generalized additive models. Additional file 1: Fig. S1c displays biological replicates from various experiments. **e** Transmission electron micrographs of cross-sections of an L3 larva of *daf-2* grown at 15 °C, a *daf-2* dauer larva grown at 25 °C, and a *daf-2;daf-12* arrested larva grown at 25 °C. Gut lumen: central panels and large rectangles in upper panels. Cuticle: lower panels and small rectangles in upper panels. Lipid droplets: arrowheads. Dauer-specific alae: arrows. Striated layer of dauer cuticle: bracket. Scale bars correspond to 5 μm (upper panels), 1 μm (central panels), and 0.5 μm (lower panels). Representative images of at least five animals per condition

The process of dauer formation is controlled by Daf genes (from dauer formation). Whereas Daf-c mutants constitutively undergo dauer arrest, Daf-d mutants are defective in forming dauer larvae. Genetic analysis of Daf mutants has revealed that dauer formation is governed by guanylyl cyclase, TGF-β-like, insulin-like, and steroid hormone signaling pathways [7, 15] (Fig. 1a). In response to changes in population density (sensed through dauer-inducing pheromones) and altered energetic metabolism (signaled by insulin-like peptides), the guanylyl cyclase, TGF-β, and insulin-like pathways converge on two transcription factors: the FoxO member DAF-16 and the nuclear hormone receptor DAF-12, both encoded by Daf-d genes that are essential for dauer formation. DAF-16 is negatively regulated by the insulin receptor homolog DAF-2 in response to stimulation by insulin-like peptides [16–18]. DAF-12, on the other hand, is governed by steroid hormones, called dafachronic acids (DAs), synthesized by the cytochrome P450 enzyme DAF-9 when the population density is low [19–22]. DAF-12 acts as a developmental switch—in its DA-bound state, it promotes reproductive development, whereas in the DA-free state, it induces dauer formation [19, 23, 24]. Because loss-of-function mutations in *daf-12* generally suppress dauer development [20], for simplicity, we refer to the dauer-promoting DA-free form as “active DAF-12.” DAF-16 and the DA-unbound DAF-12 stimulate each other but also have their separate downstream programs (Fig. 1a) [25, 26]. The interplay between these factors determines whether worms enter diapause: when both are activated, dauer formation is induced. In addition, germline-mediated crosstalk between DAF-16 and DAF-12 is essential for adult longevity [27]. Although many transcriptional and metabolic targets of DAF-16 and DAF-12 have been elucidated in the context of diapause and longevity [14, 28–37], fundamental questions remain about how these transcription factors interact to control the metabolism and what is the impact of the metabolic switch on the growth and development.

Here, we show that during dauer formation, the metabolic mode, consisting of two separately regulated modules, dictates the state of development. The insulin pathway has a dual effect that requires AMP-activated protein kinase (AMPK) activity. On the one hand, it maintains low catabolism (first module). On the other hand, it acts together with the steroid hormone pathway to inhibit the TCA cycle and promote gluconeogenesis (second module). Simultaneous inactivation of the AMPK and steroid hormone pathways leads to a switch from gluconeogenesis to an active TCA cycle via tight control of the molar ratios of competing enzymes. This metabolic transition is a prerequisite for the organism to enter into reproductive growth.

## Results

### DAF-16 induces a switch to low metabolic rate whereas DAF-12 controls dauer morphogenesis

To distinguish which signaling pathways control metabolism in the dauer state, we investigated the metabolic activities of wild-type dauers, as well as of mutants of key dauer regulatory factors. Dauer larvae have substantially diminished metabolic rate and heat production compared to other larval stages [10]. For that reason, we first examined the heat flow produced by wild-type worms undergoing reproductive development or dauer formation from the L1 larval stage onwards using time-resolved isothermal microcalorimetry. To induce a synchronous dauer formation, we grew worms on 4-methylated sterol (4-MS), which blocks the production of DAs [26]. After an initial increase of heat flow by both groups, the two trends diverged after about 24 h, increasing further in worms in the reproductive mode, while decreasing in animals that underwent dauer formation (Fig. 1b and Additional file 1: Fig. S1a). A similar trend was observed in the TGF-β Daf-c mutant *daf-7*, which forms dauer larvae but reproduces when DA is added [19, 38] (Additional file 1: Fig. S1b).

Next, we set out to determine how the insulin and steroid hormone pathways contribute to the switch. To disentangle the pathways, we chose conditions under which DAF-16 is active but not DAF-12. We made use of a group of Daf-c alleles of *daf-2*, designated class II, that is not fully suppressed by Daf-d mutations of *daf-12* or by the addition of DAs [19, 25]. One such allele is *daf-2(e1370)*. Worms bearing this mutation reproduce at the permissive temperature of 15 °C, whereas at the restrictive temperature of 25 °C, they form dauers [25]. We compared this strain to a double mutant *daf-2(e1370);daf-12(rh61rh411)* or DA-treated *daf-2(e1370)* that arrest the development at an L3-like larval stage at 25 °C due to DAF-16 activity (Fig. 1c) [19, 25]. The metabolism and morphology of these larvae have not been characterized in detail. Unlike *daf-7* on DA, at 25 °C, *daf-2;daf-12* and *daf-2* on DA shifted the metabolic mode to low heat production after 24 h (Fig. 1d and Additional file 1: Fig. S1b and S1c). In contrast, a double mutant *daf-2(e1370);daf-16(mu86)* that undergoes reproductive development at 25 °C [25] displayed high heat production (Fig. 1d and Additional file 1: Fig. S1c). Thus, DAF-12 is not required for the switch to low metabolic activity during dauer formation when DAF-16 is active.

We further asked whether DAF-16 or DAF-12 determines the morphology of dauer larvae and whether metabolic state and morphology are interconnected. Our previous studies indicated that DAF-12 could induce morphological features of dauer larvae in the absence of DAF-16 [26]. However, it was not clear whether the activation of DAF-16 alone could promote dauer morphology. To test this, we performed electron microscopy on *daf-2;daf-12* and DA-treated *daf-2* larvae grown at 25 °C. Interestingly, similar to L3 larvae, they had a large body diameter and an elongated gut lumen with long, densely packed microvilli, and lacked characteristic features of the dauer cuticle such as alae and a striated layer (Fig. 1e and Additional file 2: Fig. S2) [39]. However, similar to dauers, *daf-2;daf-12* and DA-fed *daf-2* animals deposited numerous lipid droplets (LDs) (Fig. 1e and Additional file 2: Fig. S2). Thus, DAF-12 controls dauer morphogenesis, whereas DAF-16 has no direct influence on this process but appears to affect the dauer-associated metabolic changes that culminate in low metabolic rate and high LD accumulation.

### DAF-16 controls catabolism and, together with DAF-12, promotes a shift from TCA cycle-driven metabolism to gluconeogenesis

**Fig. 2:**
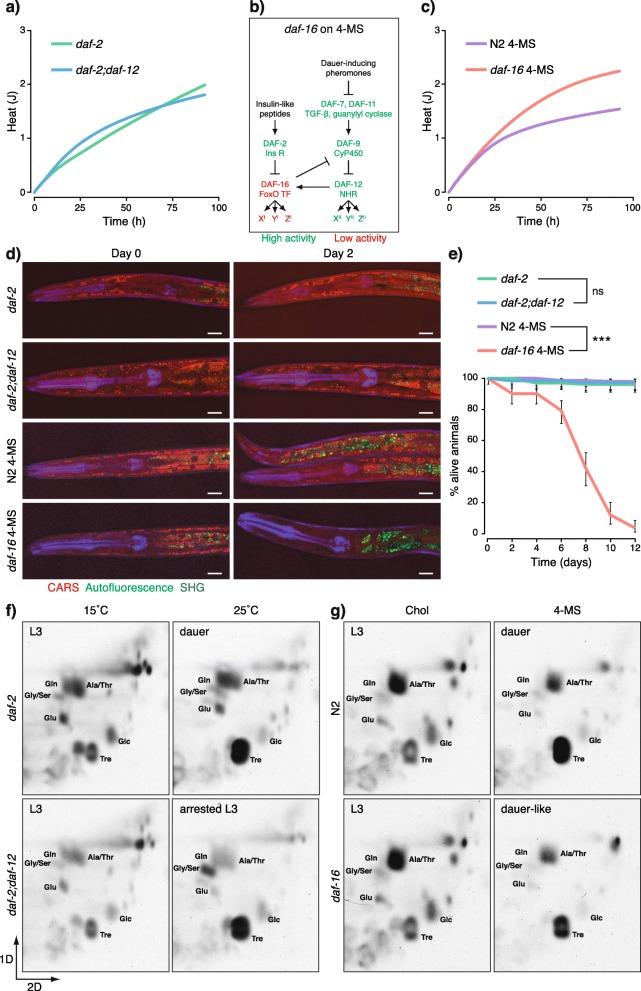
DAF-16 determines the energy expenditure and lifespan of dauer larvae and, together with DAF-12, the switch to gluconeogenesis. **a** Cumulative heat dissipation of developmentally arrested *daf-2* dauers and *daf-2;daf-12* L3 larvae. Median heat of biological replicates from one experiment. Biological replicates from various experiments are displayed in Additional file 3: Fig. S3a. **b** Formation of dauer-like larvae of *daf-16* on 4-MS. DAF-12 is activated due to a lack of DAs; however, DAF-16 activity is absent due to the mutation in the *daf-16* locus. **c** Cumulative heat dissipation of developmentally arrested wild-type (N2) dauers and *daf-16* dauer-like larvae on 4-MS. Median heat of biological replicates from one experiment. Biological replicates from various experiments are displayed in Additional file 3: Fig. S3b. **d** Coherent anti-Stokes Raman scattering (CARS) microscopy of lipid droplets in *daf-2* dauers and *daf-2;daf-12* arrested L3 larvae grown at 25 °C, and wild-type (N2) dauers and *daf-16* dauer-like larvae grown on 4-MS. SHG, second harmonic generation. Representative images of at least 6 animals, scale bars 10 μm. **e** Survival rates of *daf-2* dauers and *daf-2;daf-12* arrested L3 larvae grown at 25 °C, and wild-type (N2) dauers and *daf-16* dauer-like larvae grown on 4-MS. Means ± 95% confidence intervals of 3 experiments with 3 replicates. ***p < 0.001; ns, no significant difference determined by log-rank test. **f** 2D-TLC of ^14^C-acetate-labeled metabolites from *daf-2* dauers and *daf-2;daf-12* arrested larvae grown at 25 °C compared to L3 larvae grown at 15 °C. Representative images from at least 2 experiments. **g** 2D-TLC of ^14^C-acetate-labeled metabolites from wild-type (N2) dauers and *daf-16* dauer-like larvae on 4-MS compared to L3 larvae on cholesterol. Representative images from at least 2 experiments. **f**, **g** Tre, trehalose; Glc, glucose; Glu, glutamate; Gly, glycine; Ser, serine; Gln, glutamine; Ala, alanine; Thr, threonine

Cells produce heat almost exclusively through catabolic reactions [40]. Thus, low heat production in dauers indicated that they have decreased catabolism. To determine which pathways regulate this process, we compared the amounts of heat that *daf-2* dauers and *daf-2;daf-12* L3-like larvae produce after entering the arrested state. Food was omitted to exclude heat generation by bacteria. *daf-2* dauers and *daf-2;daf-12* larvae generated similar amounts of heat (Fig. 2a and Additional file 3: Fig. S3a), suggesting that DAF-16 does not require DAF-12 activity to regulate the energy expenditure. We next asked how the loss of DAF-16 activity would influence the metabolic rate. For the inactivation of DAF-16, we used *daf-16(mu86)* mutants grown on 4-MS. Under these conditions, DAF-12 promotes the dauer program, but DAF-16 is absent and worms arrest as dauer-like animals [26] (Fig. 2b). Compared to wild-type dauers, 4-MS-treated *daf-16* animals displayed higher heat production in their arrested state (Fig. 2c and Additional file 3: Fig. S3b), suggesting that DAF-16 suppresses metabolic rate and the catabolism of energy stores of dauers.

We, therefore, monitored the breakdown of lipids, sugars, and amino acids in *daf-2;daf-12* and *daf-16* on 4-MS. Because food was omitted, only the catabolism of internal energy reserves would account for any observed change. Storage triglycerides (TGs) were visualized by coherent anti-Stokes Raman scattering (CARS) microscopy of LDs and by thin-layer chromatography (TLC). *daf-2* dauers and, to a slightly lesser extent, *daf-2;daf-12* arrested larvae and wild-type dauers on 4-MS, conserved their TGs over time (Fig. 2d and Additional file 4: Fig. S4a). In contrast, *daf-16* larvae on 4-MS were depleted of TGs only after 2 days (Fig. 2d and Additional file 4: Fig. S4a). Phospholipids were preserved in all animals, suggesting that no substantial degradation of membranes occurred (Additional file 4: Fig. S4a). Furthermore, sugars and amino acids were maintained at high levels in wild-type dauers on 4-MS, *daf-2*, and *daf-2;daf-12* larvae, but were rapidly degraded in *daf-16* on 4-MS (Additional file 4: Fig. S4a and S4b). These results show that, in the absence of DAF-16, catabolism becomes misregulated and the energy depot is dramatically depleted.

The above findings suggest that faster depletion of energy reserves might reduce survival in *daf-16* larvae. Indeed, the viability of these animals declined rapidly and they perished after 12 days, while almost 100% of dauers and *daf-2;daf-12* arrested larvae remained viable (Fig. 2e). Thus, DAF-16 regulates the survival of dauer larvae by controlling energy expenditure.

The switch to a lower catabolic rate in dauers is accompanied by a shift from TCA cycle-driven metabolism to gluconeogenesis, leading to the accumulation of the disaccharide trehalose [9]. To determine whether DAF-16 or DAF-12 is responsible for this transition, we used 2D-TLC to trace the metabolism of ^14^C-radiolabeled acetate. Carbon atoms of acetate are only incorporated into trehalose if the glyoxylate shunt and the gluconeogenesis are active [9]. This labeling strategy mimics the usage of endogenous lipids as a carbon source for gluconeogenesis because both the lipid catabolism and the external acetate provide acetyl-CoA that enters the TCA or the glyoxylate cycle. As shown before [9], *daf-2* dauers at 25 °C displayed stronger accumulation of labeled trehalose than L3 larvae at 15 °C (Fig. 2f). High incorporation of acetate into trehalose was also observed in *daf-2;daf-12* arrested L3 larvae (Fig. 2f). Thus, activation of DAF-16 is sufficient to trigger gluconeogenesis. Surprisingly, we also detected higher levels of labeled trehalose in *daf-16* mutants cultured on 4-MS, suggesting that DAF-12 can promote a gluconeogenic mode in the absence of DAF-16 (Fig. 2g). Besides, we noticed that all developmentally arrested stages displayed lower levels of a band corresponding to the amino acids alanine and threonine compared to the related L3 larvae (Fig. 2f, g). Because these amino acids can be derived from glycolysis/gluconeogenesis and the TCA/glyoxylate cycle (from pyruvate and oxaloacetate, respectively), we concluded that their synthesis might be downregulated redundantly by DAF-16 or DAF-12 to ensure higher availability of substrates for gluconeogenesis. In this context, DAF-16 may exert more stringent regulation because animals bearing *daf-2(e1370)* showed lower levels of these compounds in both growing and arrested state compared to the wild-type or *daf-16* worms. It is known that even at the permissive temperature, DAF-16 is nuclear in *daf-2(e1370)* but not in the wild-type worms [18], suggesting that this trait may be transcriptionally promoted by DAF-16.

Together, our results demonstrate that DAF-16 alone maintains low catabolism, whereas DAF-16 and DAF-12 separately promote a shift from TCA cycle-driven metabolism to gluconeogenesis. Besides, the low catabolism and the gluconeogenic mode are independent metabolic modules that can be uncoupled under conditions of low DAF-16 but high DAF-12 activity.

### AAK-2 is required for the DAF-16-mediated metabolic switch and developmental arrest

**Fig. 3:**
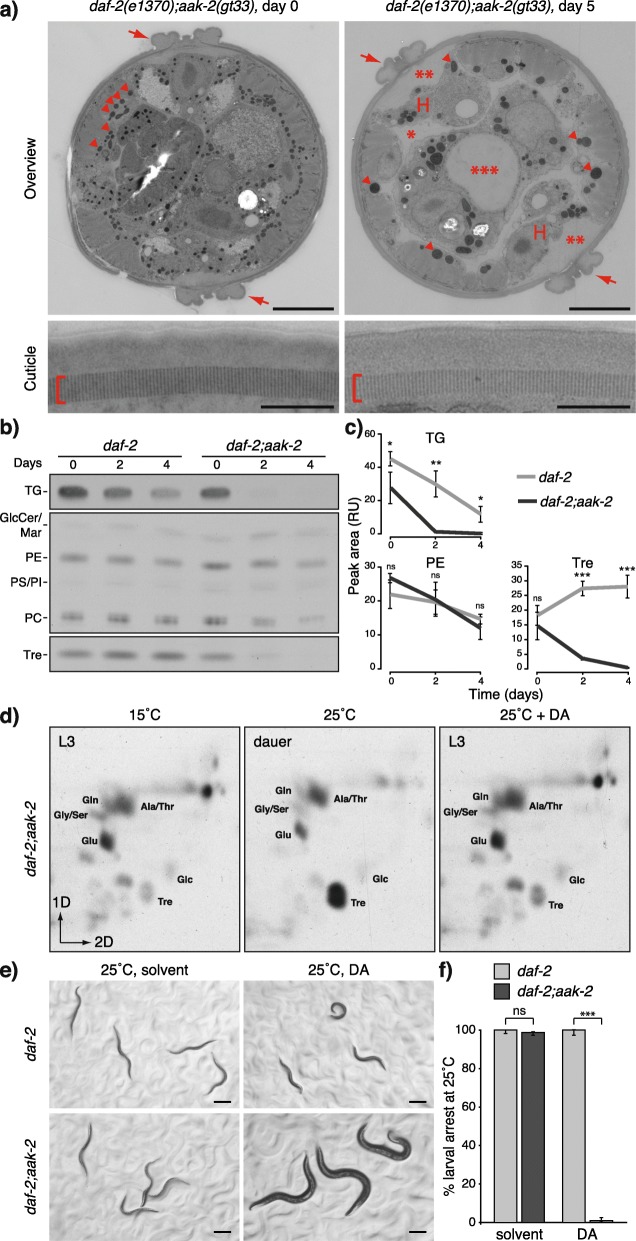
AAK-2 regulates the catabolism, the gluconeogenic mode, and the developmental arrest in the dauer state. **a** Electron microscopy of *daf-2(e1370);aak-2(gt33)* collected on the day of the dauer formation or after 5 days of incubation at 25 °C on ample food source. Alae: arrows. Striated layer of the cuticle: brackets. Mitochondria: arrowheads. Starvation features: shrinkage of the hypodermis (H), expansion of the pseudocoelomic cavity (1 asterisk), a cavity between the cuticle and the hypodermis (2 asterisks), widening of the gut lumen (3 asterisks). Scale bars 5 μm (upper panels) and 0.5 μm (lower panels). Representative images of at least 4 animals. **b** TLC of ^14^C-acetate-labeled lipids and sugars from *daf-2* and *daf-2;aak-2* dauers measured at different time points after the arrest. TG, triglycerides; GlcCer, glucosylceramides; Mar, maradolipids; PE, phosphatidylethanolamines; PS, phosphatidylserines; PI, phosphatidylinositols; PC, phosphatidylcholines; Tre, trehalose. **c** Quantification of band intensities in some of the compounds in **b** represented by the peak area of the optical density in relative units (RU). Means ± SD of 2 experiments with 3 biological replicates. ***p < 0.001; **p < 0.01; *p < 0.1; ns, no significant difference determined by Student *t* test. **d** 2D-TLC of ^14^C-acetate-labeled metabolites from *daf-2;aak-2* L3 larvae grown at 15 °C, dauers grown at 25 °C, and L3 larvae obtained at 25 °C in the presence of DA. DA, dafachronic acid; Tre, trehalose; Glc, glucose; Glu, glutamate; Gly, glycine; Ser, serine; Gln, glutamine; Ala, alanine; Thr, threonine. Representative images from at least 2 experiments. **e** Micrographs of *daf-2* and *daf-2;aak-2* animals grown at 25 °C in the presence or absence of DA. **f** Quantification of the larval arrest in **e**. Means ± 95% confidence intervals of 3 experiments with 3 replicates. ***p < 0.001; ns, no significant difference determined by one-way analysis of variance

We postulated that under conditions of high DAF-16 but low DAF-12 activity (Fig. 1c), the disruption of a hypothetical factor required for the DAF-16-mediated metabolic switch could promote higher catabolism and prevent the gluconeogenic mode. Moreover, if the metabolic and developmental transitions are coupled, one prediction would be that such an intervention may rescue the developmental arrest caused by DAF-16. Thus, it was of high importance to identify such a factor. The uncontrolled catabolism and mortality in *daf-16* on 4-MS were very similar to those observed in dauers with loss of activity of the AMPK α-subunit AAK-2 [41]. Thus, DAF-16 and AAK-2 may jointly control the metabolic state of dauers, making AMPK a potential candidate for this factor. We first asked whether *aak-2* mutant dauers lose TGs, sugars, and amino acids similar to *daf-16* on 4-MS. We generated a *daf-2(e1370);aak-2(gt33)* strain harboring a large deletion in *aak-2*. At 25 °C, almost all animals formed dauer larvae with typical dauer morphology (Fig. 3a). Curiously, although a previous study that used a strain *daf-2(e1370);aak-2(ok524)*, bearing a different deletion in *aak-2*, showed that at 25 °C, the animals spontaneously exited from dauer state and produced adults within 5 days [42], dauers of *daf-2(e1370);aak-2(gt33)* grown on a solid medium with ample food for 5 days at 25 °C did not undergo spontaneous exit from dauer state. Almost all worms survived the treatment with the detergent SDS, which is a hallmark of dauer larvae [43] (Additional file 5: Fig. S5a and S5b). The dauer-specific alae and striated layer of the cuticle were also preserved over time (Fig. 3a). Hence, using *daf-2(e1370);aak-2(gt33)* is a very suitable model to study the metabolic control in the dauer state.

Electron micrographs suggested that after 5 days, *daf-2;aak-2* dauers enter a state of starvation characterized by an extreme decrease of the cellular volume of the hypodermis, expansion of the body cavities, and deterioration of the mitochondria (Fig. 3a). In line with these observations, TGs and trehalose (Fig. 3b, c), as well as amino acids (Additional file 5: Fig. S5c), were rapidly depleted. Phospholipids were less affected (Fig. 3b, c). To test the cellular response to starvation in AMPK mutants, we monitored FIB-1, a small nucleolar ribonucleoprotein (snoRNP) whose localization to the nucleolus is reduced during starvation or loss of TOR activity [44]. Dauers without Daf mutations isolated from overcrowded plates had nucleolar FIB-1 consistent with a non-starved state (Additional file 5: Fig. S5d). *daf-2* and *daf-2;aak-2* dauers, as well as *daf-2* DA-fed larvae, also displayed nucleolar FIB-1 shortly after the arrest (Additional file 5: Fig. S5e). This localization remained unchanged over time in *daf-2* dauers and *daf-2* DA-fed arrested L3 larvae (Additional file 5: Fig. S5e). In *daf-2;aak-2* dauers, however, FIB-1 formed granular structures in the nucleoplasm after 4 days and was almost completely dispersed in the nucleoplasm of cells after 7 days (Additional file 5: Fig. S5e). Thus, *daf-16* and *aak-2* mutants have highly related phenotypes in terms of catabolism in the dauer state and may act in the same pathway. Moreover, DAF-16 could prevent a TOR-dependent starvation response in the absence of DAF-12, but not of AAK-2, suggesting that AMPK might be required for the DAF-16-mediated maintenance of the energy reserves.

Since the disruption of *aak-2* enhanced catabolism in *daf-2* dauers, we asked if it could also abolish the gluconeogenic mode. ^14^C-acetate labeling in *daf-2;aak*-2 at 25 °C showed a pronounced gluconeogenic mode (Fig. 3d). Because DAF-12 could activate gluconeogenesis in the absence of DAF-16, we asked whether it could perform this activity also in the absence of AAK-2. Remarkably, when we inhibited DAF-12 in *daf-2;aak-2* by adding DA, the gluconeogenesis was abolished (Fig. 3d). Furthermore, *daf-2;aak-2* showed higher glycine/serine levels at 15 °C and 25 °C with DA and lower at 25 °C without DA (Fig. 3d). Since glycine can be produced from glyoxylate, and serine from the glycolysis/gluconeogenesis intermediate 3-phosphoglycerate, the production of these amino acids from acetate may be kept low by AAK-2 and DAF-12 so that glyoxylate and 3-phosphoglycerate are more efficiently used in trehalose synthesis. Hence, AAK-2 fulfills the criteria for a factor required for the switch to low energy expenditure and to gluconeogenesis induced by DAF-16 when DAF-12 is inhibited. To determine whether AAK-2 is also necessary for the DAF-16-induced growth arrest, we monitored the development of *daf-2;aak-2* worms at 25 °C with DA. Astoundingly, these worms completely bypassed dauer arrest and developed into adults (Fig. 3e, f). Thus, in *daf-2* mutants, AAK-2 is essential for the DAF-16-mediated growth and metabolic transition in the absence of DAF-12 activity.

An interaction between *daf-2* and *aak-2* has also been observed in the context of adult longevity: the lifespan extension characteristic for *daf-2* animals is fully suppressed by *aak-2* mutations [45]. Thus, the metabolic mode associated with increased longevity in adult *daf-2* mutants [46] could depend on AAK-2. To assess the gluconeogenesis, we labeled *daf-2* and *daf-2;aak-2* with ^14^C-acetate and grew them at 15 °C until the L4 stage to bypass dauer formation. From this point on, we either kept them at 15 °C to maintain the DAF-2 activity high or shifted them to 25 °C to suppress DAF-2. This temperature shift doubles the lifespan of *daf-2* adults compared to the wild-type worms [47]. Twenty-four hours later, we extracted the metabolites and observed much higher accumulation of labeled trehalose in *daf-2* worms at 25 °C as compared to 15 °C (Additional file 5: Fig. S5f). *daf-2;aak-2* displayed lower incorporation of ^14^C-acetate into trehalose at both 15 °C and 25 °C in comparison with *daf-2.* This observation shows that AAK-2 is required for the full extent of the metabolic switch in *daf-2* adults. A small elevation of labeled trehalose in *daf-2;aak-2* at 25 °C compared to 15 °C (Additional file 5: Fig. S5f) suggests that in adults, DAF-16 could also promote gluconeogenesis to a very limited degree in an AAK-2-independent manner. One limitation of our study is that the 2D-TLC does not allow for quantitative evaluation of intermediate phenotypes such as the one observed in adult worms of *daf-2;aak-2* at 25 °C. Future studies will be required to evaluate the exact flux through the metabolic pathway. Based on our observations, we conclude that the metabolic switch does not only determine dauer diapause but is also activated in adult worms with reduced insulin signaling and correlates with the lifespan extension typical for them.

### Gluconeogenesis is turned on by a shift in the molar ratios of key metabolic enzymes

To gain insight into the molecular mechanism underlying the switch to gluconeogenesis and how AAK-2 and DAF-12 control it, we employed the LC-MS/MS method of MS Western [48] to quantify the absolute (molar) amount of 43 individual enzymes or subunits of enzymatic complexes involved in TCA cycle, glyoxylate shunt, glycolysis, gluconeogenesis, and mitochondrial pyruvate metabolism. The molar abundance of each protein was determined by comparing individual abundances of several (typically, 2 to 5) quantitypic peptides with ^13^C, ^15^N-isotopically labeled peptide standards (Additional file 6: Fig. S6a-f). Standard peptides were concatenated into a protein chimera (Additional file 6: Fig. S6g) that was in-gel co-digested with target proteins separated by one-dimensional SDS-PAGE from a whole animal lysate. The molar amount of chimera protein was referenced to the standard of BSA and quantified in the same LC-MS/MS experiment. MS Western quantification was highly concordant. The median coefficient of variation of molar abundances of proteins determined using alternative standard peptides was less than 10% (Additional file 6: Fig. S6h) with better than 0.99 Pearson coefficient of correlation between technical replicas (Additional file 6: Fig. S6i). The molar abundances of individual proteins were normalized to the total protein content in each animal lysate and could be directly compared between all biological conditions without metabolic or chemical labeling of target proteins.

**Fig. 4:**
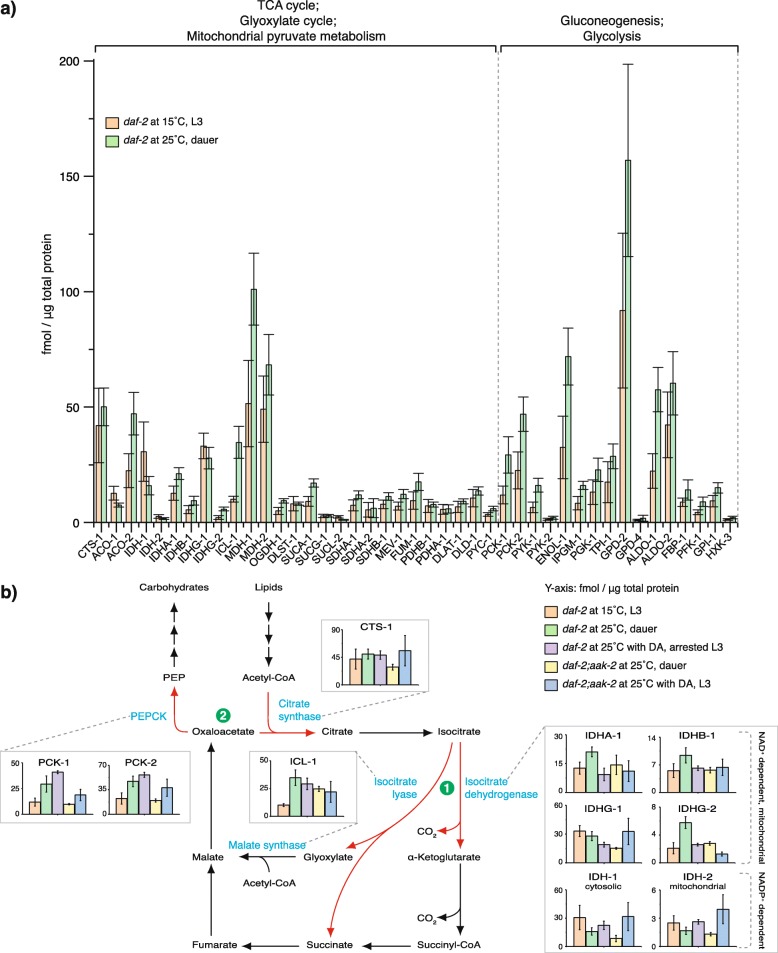
The metabolic switch is achieved through the regulation of enzymes that work on branch points between competing pathways. **a** Absolute quantification of 43 enzymes of the TCA and glyoxylate cycle, mitochondrial pyruvate metabolism, gluconeogenesis, and glycolysis in *daf-2* dauers grown at 25 °C compared to *daf-2* L3 larvae at 15 °C. Means ± standard deviation of 3 biological replicates with 2 technical replicates each. **b** Schematic representation of the pathway that converts lipids to carbohydrates with absolute quantification of the enzymes that operate at the branch points in *daf-2* L3 larvae at 15 °C and animals of *daf-2* and *daf-2;aak-2* grown at 25 °C with or without DA. Red arrows and green circles represent the competing reactions at the point of divergence between (1) TCA and glyoxylate pathway and (2) the recycling of oxaloacetate into the TCA/glyoxylate cycle or its entry into gluconeogenesis. Means ± standard deviation of 3 biological replicates with 2 technical replicates each. Additional file 9: Tab. S1 contains the molar abundances of all 43 proteins in biological replicates for all tested conditions

We first analyzed the enzyme levels in dauers (*daf-2* at 25 °C) and L3 larvae (*daf-2* at 15 °C). The 43 enzymes were detected in a wide range of 1 to nearly 160 fmol/μg of the total protein (Fig. 4a, Additional files 7, 8, and 9: Fig. S7, S8a, and Tab. S1). Although a global metabolic perturbation would be expected, we found that the balance of molar abundances of members of different pathways between the two groups was not perturbed. However, in dauers, the enzymes of glycolysis/gluconeogenesis were slightly more prevalent with respect to other pathways indicating enhanced gluconeogenesis (Additional file 8: Fig. S8b). Interestingly, in total, dauers were 1.7-fold more enriched in metabolic enzymes compared to L3 larvae, despite that dauer is a metabolically reduced stage (Additional file 8: Fig. S8c). Glycolysis/gluconeogenesis enzymes were enriched to a greater extent in dauers compared to L3 larvae (Additional file 8: Fig. S8d). Hence, the overall architecture of the metabolic network was preserved in both developmental conditions, while the metabolic switch was executed by fine-tuning its directionality.

To understand the metabolic switch mechanism, we reconstructed the pathway that converts lipid-derived acetyl-CoA to carbohydrates via glyoxylate shunt and gluconeogenesis (Fig. 4b, Additional files 7 and 8: Fig. S7 and S8a). We focused on several reactions that act as branch points or “metabolic turnouts.” The first set of reactions determines whether acetyl-CoA, via isocitrate, will enter the TCA or the glyoxylate cycle (Fig. 4b and Additional file 7: Fig. S7). We observed 3.5-fold upregulation of the glyoxylate cycle enzyme ICL-1 in dauers compared to L3 larvae (Fig. 4b and Additional file 7: Fig. S7). This was consistent with previous studies [29, 46] and suggested that dauers have higher glyoxylate pathway activity. Thus, they convert isocitrate to malate and succinate without losing carbon atoms in the TCA cycle via decarboxylation (Fig. 4b and Additional file 7: Fig. S7). Whether these carbon atoms are used for gluconeogenesis depends on the reactions at the second branch point. This point will determine whether the oxaloacetate produced downstream of the glyoxylate pathway is recycled by the citrate synthase or converted to phosphoenolpyruvate (PEP) by phosphoenolpyruvate carboxykinase (PEPCK) (Fig. 4b and Additional file 8: Fig. S8a). The production of PEP by PEPCK is the first and pathway-specific step of gluconeogenesis. Similar to ICL-1, the two isoforms of PEPCK, PCK-1 and PCK-2, were 2-fold elevated in *daf-2* dauers compared to L3 larvae (Fig. 4b and Additional file 8: Fig. S8a). Thus, dauers have a higher ability to use acetyl-CoA for gluconeogenesis. This ability is further supported by ~ 2-fold increase of oxaloacetate-producing malate dehydrogenase (MDH-1) and enzymes shared between gluconeogenesis and glycolysis such as enolase (ENOL-1) and aldolase (ALDO-1) (Fig. 4a, Additional files 7 and 8: Fig. S7 and S8a).

**Fig. 5:**
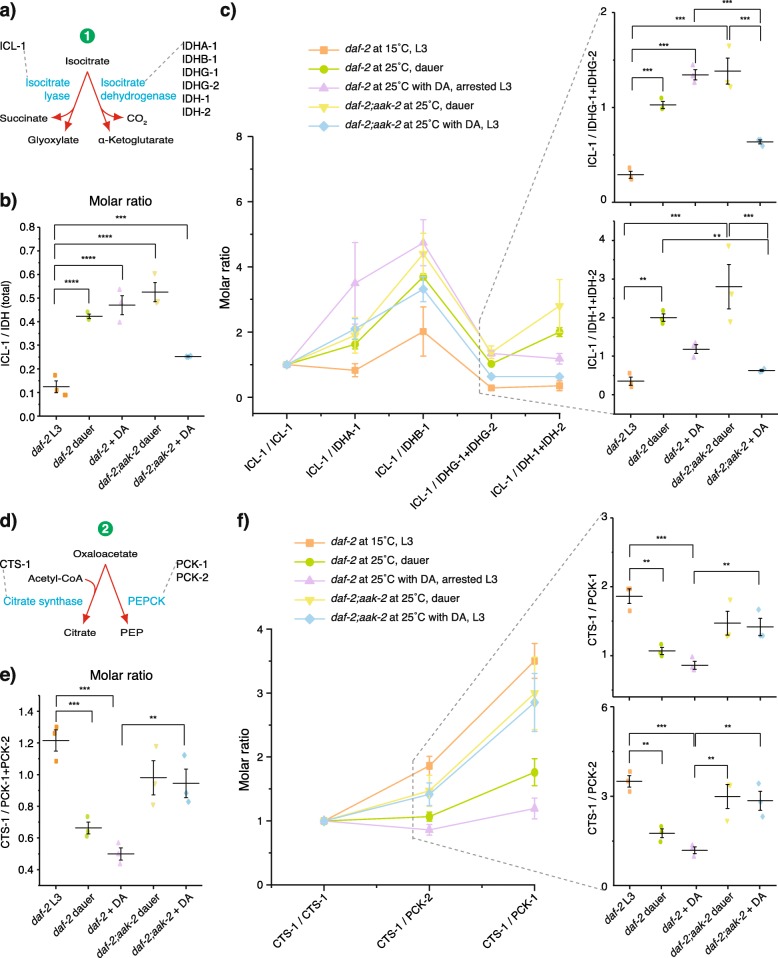
DAF-12 and AAK-2 control the molar ratios of the enzymes at the branch points between competing pathways. **a** Scheme of the first branch point—the entry of isocitrate into glyoxylate or TCA cycle. **b** Molar ratio between ICL-1 and the summed abundances of all isocitrate dehydrogenase isoforms and subunits, IDHA-1, IDHB-1, IDHG-1, IDHG-2, IDH-1, IDH-2, dubbed IDH (total). **c** Molar ratios between ICL-1 and individual isocitrate dehydrogenases. The sums of IDHG-1+IDHG-2 and IDH-1+IDH-2 are provided as a clearer representation due to the low molar abundance of IDHG-2 and IDH-2 compared to IDHG-1 and IDH-1, respectively. Lines between data points are provided for better visualization. **d** Scheme of the second branch point—the recycling of oxaloacetate into citrate or its entry into gluconeogenesis. **e** Molar ratio between CTS-1 and the summed abundances of the two PEPCK isoforms PCK-1 and PCK-2. **f** Molar ratio between CTS-1 and the individual PEPCK isoforms. Lines between data points are provided for better visualization. In all panels, means ± SD of 3 biological replicates with 2 technical replicates each; *p* values represent *p* > 0.05 (ns), *p ≤ 0.05, **p ≤ 0.01, ***p ≤ 0.001, ****p ≤ 0.0001. One-way ANOVA was performed with Holm-Bonferroni statistical method

As shown above, simultaneous inactivation of DAF-12 and AAK-2 in *daf-2* at 25 °C prevents the gluconeogenic mode and the developmental arrest (Fig. 3d–f). Thus, we asked whether DAF-12 or AAK-2 is responsible for the altered expression of the enzymes. To test this, we quantified all 43 enzymes in *daf-2* on DA and *daf-2;aak-2* with or without DA at 25 °C. We first analyzed the TCA/glyoxylate cycle branch point. As expected, larvae with high gluconeogenic mode (*daf-2* on DA and *daf-2;aak-2* without DA) showed similar upregulation of ICL-1 as in *daf-2* dauers (Fig. 4b and Additional file 7: Fig. S7). Interestingly, *daf-2;aak-2* on DA also had higher amounts of this enzyme despite the low gluconeogenic mode (Fig. 4b and Additional file 7: Fig. S7). We reasoned that not only the absolute levels of ICL-1 but its molar ratio with respect to competing TCA cycle enzymes (isocitrate dehydrogenases) controls the isocitrate flow into the glyoxylate shunt (Fig. 5a). Indeed, ICL-1 displayed the lowest molar ratio to all isocitrate dehydrogenase subunits and isoforms in *daf-2* L3 larvae at 15 °C (Fig. 5b, c). This was consistent with a more intensive TCA cycle. The ratios were overall higher in *daf-2* and *daf-2;aak-2* at 25 °C with or without DA (Fig. 5b, c). However, in stages with pronounced gluconeogenic mode (*daf-2* without or with DA at 25 °C, *daf-2;aak-2* at 25 °C), this elevation was much more pronounced (3.5-, 3.8-, and 4.2-fold) compared to *daf-2;aak-2* at 25 °C with DA (2-fold, Fig. 5b). In DA-treated *daf-2;aak-2*, ICL-1 was much less dominant in respect to the IDHG-1 subunit of the NAD^+^-dependent isocitrate dehydrogenase and, importantly, to the NADP^+^-dependent IDH-1, which has been implicated in the regulation of DA signaling [14] (Fig. 5c, Additional file 8: Fig. S8e and S8f). Thus, simultaneous inactivation of DAF-12 and AAK-2 lowers the capacity of the glyoxylate shunt.

We next asked whether DAF-12 or AAK-2 controls the production of PEP by PEPCK at the second branch point. DA-treated *daf-2* at 25 °C had similarly elevated PCK-1 and PCK-2 as *daf-2* dauers (Fig. 4b and Additional file 8: Fig. S8a). The AAK-2-deficient strain, however, showed low PCK-1 and PCK-2 levels regardless of the presence or absence of DA (Fig. 4b and Additional file 8: Fig. S8a). This indicated that AAK-2 might promote the conversion of oxaloacetate to PEP (Fig. 5d). Accordingly, *daf-2* at 25 °C with or without DA showed lower molar ratios of citrate synthase to PEPCK, whereas in *daf-2;aak-2* at 25 °C with or without DA, they were much higher (Fig. 5e, f). It is, therefore, possible that the gluconeogenic mode in *daf-2;aak-2* at 25 °C without DA is maintained through higher recycling of oxaloacetate through the glyoxylate shunt. Future analysis will determine the precise metabolic fluxes in these animals.

Enzyme stoichiometry may also influence the flux through bidirectional reactions that are catalyzed by separate enzymes in the opposite directions if the molar ratios between these enzymes change. One such step, the rate-limiting interconversion of fructose 6-phosphate and fructose 1,6-bisphosphate in glycolysis and gluconeogenesis, largely determines the balance between the two pathways and is under the control of multiple metabolic regulators including AMPK [49]. However, when we compared L3 and dauer larvae, we did not detect significant changes in the molar ratio between the glycolytic enzyme PFK-1 and the gluconeogenesis-specific enzyme FBP-1 which are responsible for these reactions (Additional file 8: Fig. S8g). A limitation of our study is the lack of information on the post-translational modifications of the enzymes that would complement the absolute molar quantification. However, we can conclude that in such reactions, most probably, conserved regulation mechanisms via post-translational and allosteric control take place.

Together, our results suggest that, via control of molar ratios of enzymes, both DAF-12 and AAK-2 could induce the switch from TCA to glyoxylate cycle, while AAK-2 promotes the entry of carbon from these cycles into gluconeogenesis. Thus, the stoichiometry of metabolic enzymes is unequivocally associated with the transition from growth to quiescence.

## Discussion

**Fig. 6:**
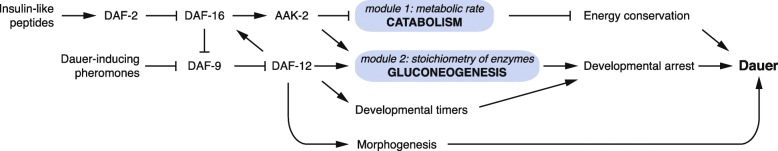
Metabolic control in the transition to the dauer state. The model represents the genetic control of metabolic and developmental determinants of dauer formation and their interactions with respect to the establishment of the dauer state. The metabolic shift consists of two modules—module 1 comprises the overall metabolic rate, mainly reflecting the catabolism of energy reserves, and module 2 affects the stoichiometry of metabolic enzymes and, thus, the directionality of metabolic pathways. DAF-16 and AAK-2 inhibit catabolism and promote energy conservation required for the long-term survival of dauers. In parallel to DAF-12, they also promote a shift in the stoichiometry of metabolic enzymes that underlies the enhanced gluconeogenesis and stimulates developmental arrest. The latter occurs at the third larval stage due to the activity of developmental timers, controlled at least partly by DAF-12. The metabolic and physiologic adaptations for survival are complemented by the specific morphogenetic program under the control of DAF-12

This study demonstrates that the transition between growth and quiescence, as well as the mode of metabolism in long-lived insulin-signaling mutants, depends on a metabolic switch. Our results provide insight into how major signaling pathways govern the metabolic transition, consisting of two separately controlled modules (Fig. 6). In dauer formation, DAF-16 and AAK-2 inhibit catabolism (module 1) and promote energy conservation, required for the long-term survival of dauers. In parallel, DAF-16, AAK-2, and DAF-12 stimulate gluconeogenesis (module 2) of compounds produced by the glyoxylate pathway. This favors the synthesis of substrates for glycolysis and lowers the TCA cycle and the production of building blocks for anabolism. Consequently, worms cease growth and enter a long-lasting dauer state. When executed in adults, this metabolic mode may contribute to an extension of the lifespan by preventing pathologies associated with activation of growth/reproductive programs during the post-reproductive period [50].

Knowing the molar amounts of enzymes involved in the metabolic switch (in contrast to the fold changes between two or more biological conditions) was instrumental in differentiating the contributions of signaling pathways in the metabolic control. Simultaneous absolute quantification of 43 metabolic enzymes with excellent accuracy and precision was enabled by the method of MS Western that outperforms the conventional Western blotting by the molecular specificity, scope of target proteins, and dynamic range [48]. By building the first quantitative map of key metabolic enzymes in *C. elegans*, we discovered that, despite the low metabolic rate, dauer larvae maintain an intact metabolic network. Moreover, the enzyme abundance with respect to total protein mass in dauers is even higher than in reproductive larvae. Since catabolic activity and the switch to gluconeogenesis are co-regulated, these findings demonstrate that the apparent decrease in metabolic activity is not achieved through a decrease in the capacity of the metabolic network, but at least partly through suppression of catabolism. The metabolic switch is achieved through adjustments of the enzyme ratios rather than by some on-off mechanism, in which (at least some) enzymes are absent or overproduced. Once put into a favorable environment, only small adjustments to the steady-state levels of the key enzymes that constitute the branch points would be necessary to respond to favorable environmental cues. The dauer could then immediately use the available constellation of metabolic enzymes to support reproductive development with no need to re-synthesize them at the cost of massive expenditure of energy.

The exact molar ratios between enzymes acting as metabolic turnouts demonstrated that AAK-2 and DAF-12 equally promoted switching from TCA to glyoxylate cycle, whereas AAK-2 was the primary factor controlling the exit of substrates from the glyoxylate pathway towards gluconeogenesis. Importantly, the combined regulation of the reactions that constitute the two branch points makes the metabolic switch more robust. This is demonstrated by the fact that *daf-2;aak-2* without DA are in gluconeogenic mode despite the high citrate synthase to PEPCK molar ratio. Conceivably, the higher capacity of the glyoxylate shunt in these animals promoted by DAF-12 prevents the carbon from escaping through decarboxylation in the TCA pathway. This leads to higher recycling of oxaloacetate that, although with a lower rate, could enter into gluconeogenesis. However, when DAF-12 is inhibited in *daf-2;aak-2* by addition of DA, the oxaloacetate is not recycled to the same extent and ultimately becomes a substrate of the TCA cycle. Thus, in animals with simultaneously disrupted AAK-2 and DAF-12 signaling, the gluconeogenic mode is not occurring. In addition, the fact that the AMPK and steroid hormone pathways independently govern the switch and have different effects on it shows that the metabolic control is fine-tuned through the integration of signaling pathways that work in parallel.

An important question is how the information about the state of metabolism is translated into signals that influence growth and development. Most probably, signaling cascades are sensitive to steady-state concentrations of key metabolites or to fluxes through given pathways. In line with this, we have previously shown that the dauer developmental decision is fine-tuned through regulation of the levels of NADPH required for DA synthesis [14]. This NADPH is generated in reactions directly related to the gluconeogenic mode: the oxidative steps of the pentose phosphate pathway (PPP) and the oxidative decarboxylation of isocitrate by the abovementioned NADP^+^-dependent isocitrate dehydrogenase IDH-1 [14]. The establishment of the gluconeogenic leads to a reduction of the substrates for NADPH production: the synthesis of trehalose consumes glucose 6-phosphate required for PPP [14], while the glyoxylate pathway competes for isocitrate needed for the IDH-1 reaction. Consequently, when worms switch to gluconeogenic mode, the NADPH levels, and thus, the production of DA, is diminished. In the context of the present study, this suggests that the state of gluconeogenesis induces a feedback regulation on the dauer signaling via the steroid hormone pathway. Thus, the second metabolic module directly influences the developmental arrest (Fig. 6). We can predict that future studies will discover further metabolites that influence development.

We also show that metabolic mode and morphology are independently regulated in the dauer state (Fig. 6), since the arrested larvae of *daf-2;daf-12* and DA-treated *daf-2* mutants display dauer metabolism but reproductive morphology. Among several forms of postembryonic diapause in *C. elegans* (such as L1, L3, L4, and adult reproductive diapause) [1, 51–53], the dauer state is morphologically the most distinct one. Therefore, it is plausible that a conserved metabolic program is underlying the metabolic mode of all diapause stages, including the dauer larva. The other forms of diapause may, in this context, represent alternative models of uncoupling of the diapause metabolism and developmental arrest from specific diapause-related morphology.

Another challenge will be to understand how the metabolic switch is coordinated with developmental timing. During growth arrest, cells must simultaneously undergo metabolic depression and acquire dauer-specific fates. Therefore, the master regulators of dauer formation, DAF-12 and DAF-16, should also regulate cell fate decisions. Indeed, DAF-12 has been shown to suppress the progression through larval stages and to determine the cell fates via microRNAs of the *let-7* family serving as developmental timers [15] (Fig. 6), suggesting that it functions as both a metabolic and a developmental “turnout.” However, the fact that the timing of the arrest in the third larval stage is also maintained in the absence of DAF-12 activity suggests that insulin signaling has similar input via an unknown mechanism. Hence, the investigation of how the synchronization of the metabolic and the developmental programs is achieved will be an important subject for future studies.

Our finding that AMPK modulates the effect of insulin pathway signaling in the control of metabolic mode and growth resolves a long-standing problem in the field. Since class II *daf-2* alleles cannot be rescued by *daf-12* Daf-d mutations or addition of DA [19, 25], it was postulated that an unknown factor mediates larval arrest in worms with active DAF-16 but inactive DAF-12 [25]. Our data indicate that this factor is AAK-2, showing that AMPK signaling has a much broader impact on dauer development and metabolism than previously reported. In addition to the known effects of the AMPK on the regulation of energy reserves [41, 54, 55], we provide evidence that in dauers, it also determines the activities of the core metabolic pathways: TCA cycle, gluconeogenesis, glycolysis, and amino acid catabolism. Thus, in a broader context, AMPK in *C. elegans* is not only a modulator of the energy metabolism and mitochondrial function in the responses to metabolic stress [56–61] but also couples the insulin-dependent developmental decisions with the cellular energetic status and nuclear hormone receptor signaling. This notion is consistent with the work of other groups, showing that in the regulation of adult lifespan, AMPK acts at least partially downstream of DAF-16 [62] and *aak-2* loss-of-function alleles suppress the greater lifespan extension typical for *daf-2* mutants [45]. Indeed, our observation that AAK-2 is required for the full extent of the metabolic switch in long-living adults provides an explanation of how the insulin-AMPK interaction promotes longevity, at least partly through control of the metabolism. Moreover, the FoxO-AMPK-nuclear hormone receptor axis of metabolic regulation might be conserved [63, 64], suggesting that the balance between growth and quiescence could be regulated by similar mechanisms in higher organisms.

## Conclusion

Taken together, our findings indicate that diapause, as well as the lifespan extension, is ultimately connected to the combined regulation of metabolic rates and growth, thus highlighting the intricate relationship between growth, development, aging, and metabolic state.

## Methods

### Material and *C. elegans* strains

Lophenol was purchased from Research Plus (Manasquan, NJ, USA), [1-^14^C]-acetate (sodium salt) from Hartmann Analytic (Braunschweig, Germany), and Dulbecco’s medium (DMEM) from Invitrogen (Karlsruhe, Germany). (25*S*)-∆^7^–DA [65–67] and lophanol were produced in the Laboratory of Prof. H.-J. Knölker. All other chemicals were from Sigma-Aldrich (Taufkirchen, Germany).

The Caenorhabditis Genetics Centre (CGC) provided the following *C. elegans* strains: N2 (Bristol strain), *daf-7(e1372)*, *daf-2(e1370)*, *daf-16(mu86)*, and *aak-2(gt33)*, *unc-119(ed3);knuSi221*. The strain *unc-119(ed3);knuSi221* contains a single-copy transgenic segment *fib-1*p*::fib-1*(genomic)::eGFP::*fib-1* 3′ UTR + *unc-119*(+). The *Escherichia coli* strain NA22 was also provided by the CGC.

The compound mutant and transgenic strains *daf-2(e1370);daf-12(rh61rh411)*, *daf-16(mu86);daf-2(e1370)*, *daf-2(e1370);aak-2(gt33)*, *daf-2(e1370);knuSi221* (abbreviated *daf-2;fib-1::eGFP*), and *daf-2(e1370);aak-2(gt33);knuSi221* (abbreviated *daf-2;aak-2;fib-1::eGFP*) were generated during this or past studies [26, 68].

### Growth and radiolabeling of *C. elegans* strains

The worm strains were routinely propagated on NGM agar plates complemented with *E. coli* NA22 [69]. When indicated, (25*S*)-∆^7^–DA was added to the bacteria to a 250 nM final concentration, calculated according to the volume of the NGM agar. The temperature-sensitive dauer constitutive strains bearing *daf-7(e1372)* or *daf-2(e1370)* alleles were propagated at 15 °C, a temperature at which they undergo reproductive growth. To obtain dauers or arrested L3 larvae of these strains, embryos were obtained from gravid adults by hypochlorite treatment [69], incubated overnight at room temperature, and the resulting synchronized L1 larvae were grown at 25 °C for 72 h.

The growth on the 4-MS-containing medium was performed according to a described method [26]. Briefly, the sterol-depleted medium was obtained by substituting agar with chloroform-extracted agarose. NA22 bacteria were grown on a sterol-free DMEM medium, pelleted, rinsed, and resuspended in M9 buffer. Two different 4-MS were used depending on the availability: lophenol or lophanol. It must be noted that these two compounds have identical effects [26]. The 4-MS (or cholesterol, when indicated) were added to the bacteria to a 13 μM final concentration according to the volume of the agarose. The worms were propagated on these plates for two consecutive generations. Again, synchronized L1 larvae were grown at 25 °C for 72 h in the second generation until the developmental arrest occurred.

For the microscopy of FIB-1::eGFP in a wild-type background, mixed populations were produced on NGM agar plates as described above. Reproductive larvae were collected from plates with abundant food and low population density. Dauer larvae were prepared from overcrowded plates and isolated from other stages by treatment with 1% SDS for 30 min followed by separation of the survived dauers from the dead debris of other stages on empty agarose plates on which the dauer larvae quickly dispersed.

To obtain radiolabeled *C. elegans*, the worms were grown on NGM agar or agarose solid medium (see above), complemented with [1-^14^C]-acetate (sodium salt). The ^14^C-acetate was added to the bacteria and calculated as 0.5 μCi/ml according to the volume of the NGM agar/agarose.

### Survival, SDS assay, and worm sampling for microscopy and biochemical analysis

Dauer(-like) and arrested L3 larvae were prepared by growing the worms on solid medium as described above, collected, and washed three times with M9 buffer. The worms were incubated in 15 ml polypropylene centrifuge tubes (Corning, NY, USA) containing 10 ml of autoclaved M9 buffer supplemented with the antibiotics streptomycin (50 μg/ml) and nystatin (10 μg/ml) at 25 °C under constant agitation. The density of the population was kept at 500 worms/ml. To monitor the survival, 100 μl aliquots were taken every 2 days and the percentage of alive animals was calculated.

For scoring the survival of *daf-2* or *daf-2;aak-2* after SDS treatment, dauer larvae were obtained by incubation at the restrictive temperature (25 °C) and subjected to treatment with 1% SDS immediately after the completion of the dauer formation (day 0) or after prolonged incubation at 25 °C (days 2 and 5) in the presence of food. After SDS treatment, the survival of the animals was scored. Separately, dauer larvae were allowed to exit by a shift to permissive temperature (15 °C) until day 2, when a mixture of reproductive stages (L3 and L4 larvae and young adults (YA)) and not recovered dauers was formed (Additional file 5: Fig. S5a). This population was also treated with SDS. Worms were collected from the feeding NGM agar plates, washed three times with ddH_2_O, and resuspended in 10 ml of 1% SDS (w/v) in ddH_2_O in 15-ml polypropylene centrifuge tubes (Corning, NY, USA). After 30 min of incubation with the SDS solution at 25 °C with shaking, the worms were washed another three times with ddH_2_O and placed on NGM agar plates where the survival was scored. Aliquots of 100 μl were also used for the preparation of microscopy samples, as described below. For biochemical analysis, the worms were washed three times with ddH_2_O, pelleted, snap-frozen in liquid nitrogen, and stored at −80 °C until further analysis (see below).

### Isothermal microcalorimetry

To measure the heat production during worm development, starting from L1 larva onwards, we first purified the eggs and plated them on agarose plates without food. After keeping them at 25 °C overnight, synchronized L1 larvae were washed with M9 buffer and diluted to 14.3 worms/μl. One hundred forty microliters (~ 2000 worms) of each suspension was pipetted into a 4 ml glass ampoule (TA Instruments, New Castle, DE, USA), in which there was already 60 μl of concentrated *E. coli* NA22 in M9 (OD_600_ = 20) so that the starting amount of bacteria was 6 OD_600_. These ampoules were then sealed with aluminum caps equipped with sealing discs (TA Instruments, New Castle, DE, USA).

For the measurements of the heat production of dauer(-like) and arrested L3 larvae, worms were grown on solid medium as described above and washed three times with M9 buffer. Two thousand larvae were collected in 200 μl of autoclaved M9 buffer supplemented with the antibiotics streptomycin (50 μg/ml) and nystatin (10 μg/ml) and transferred into 4 ml glass ampoules that were closed with aluminum caps equipped with sealing discs (TA Instruments, New Castle, DE, USA).

Isothermal calorimetric measurements were performed with a TAM III (thermal activity monitor) instrument (Waters GmbH, Eschborn, Germany) equipped with 12 microcalorimeters in twin configuration (one side for the sample, the other for a steel reference) to continuously monitor the metabolic heat produced by *C. elegans* at 25 °C for up to 5 days. The samples were held in the TAM III in a waiting position for 15 min before the complete insertion followed by 45 min equilibration. In each experiment, thermograms were recorded in replicates. The thermograms represent continuous measurements, and no curve fitting was performed.

In each experiment, the biological replicates of each group were summarized as the median heat/heat flow value at each time point across the time course of the experiment. Data points obtained after 100 h were removed. Then, these data were plotted using the ggplot2 package in R. For a better representation of the heat/heat flow patterns across time, measurement noise and technical artifacts were smoothed out with the geom_smooth function, which uses generalized additive models with integrated smoothness estimation (gam) functionality implemented in mgcv package.

### Fluorescence and CARS microscopy

For the visualization of FIB-1::eGFP by confocal microscopy and for CARS imaging of lipid deposits, worms were mounted on 2% agarose pads on glass slides (Thermo Scientific, Superfrost Plus) and anesthetized with 20 mM sodium azide in M9 buffer. The liquid was aspirated and the pads were covered with coverslips (with 0.17 ± 0.005 mm coverslips (Menzel-Glaeser)). The FIB-1::eGFP was visualized with a Zeiss LSM 880 scanning confocal microscope equipped with a LCI Plan-Neofluar 63x/1.3 Imm Korr DIC objective (Carl Zeiss Microscopy GmbH). eGFP was excited at 488 nm, and fluorescence was detected at the emission band of 490–540 nm. On average, 12 optical sections of 0.09 × 0.09 × 1 μm voxel size were collected. To represent the status of the nucleoli in all tissues within the frame, all micrographs are represented as a maximum intensity projection of the Z-stack generated in Fiji.

The imaging of lipid droplets was performed by coherent anti-Stokes Raman scattering (CARS) microscopy [70]. Autogenous two-photon excited fluorescence (TPEF) and second harmonic generation (SHG) optical signals were simultaneously acquired. TPEF was used to differentiate between lipid droplets and autofluorescent lysosome-related organelles [71]. SHG displayed collagen type I and was used as a reference for anatomical details, e.g., the position of the pharynx [72]. CARS, TPEF, and SHG were detected using a multiphoton scanning microscope coupled with two near-infrared picosecond fiber lasers. The optical microscope was an upright Axio Examiner Z.1 equipped with a laser scanning module LSM 7 (all from Carl Zeiss Microscopy GmbH, Jena, Germany) and multiple detectors in a non-descanned configuration. The excitation for TPEF and SHG was provided by an Erbium fiber laser (Femto Fiber pro NIR from Toptica Photonics AG, Gräfelfing, Germany) emitting at 781 nm with a pulse length of 1.2 ps and maximum emitted power at the source of 100 mW. The TPEF signal in the spectral range of 500–550 nm was acquired in reflection. The SHG signal was acquired in transmission mode with a bandpass (BP) filter (390 ± 9 nm). A second laser source was used to excite the CARS signal. This source (Femto Fiber pro TNIR from Toptica Photonics AG) is tunable in the range of 850–1100 nm and has a pulse length of 0.8 ps. In all CARS experiments, the wavelength was set to 1005 nm (emitted power at the source, 1.5 mW), to resonantly excite the symmetric stretching vibration of methylene groups at 2850 cm^−1^. The CARS signal was collected in transmission mode and selected using a BP filter (640 ± 7 nm). A water immersion objective W Plan-Apochromat 20×/1.0 (Carl Zeiss Microscopy GmbH) was used. Due to the transmission of optical elements, the laser power in the sample was 52 mW. CARS, TPEF, and SHG were combined as RGB images (red, CARS; green, TPEF; blue, SHG). An automatic tiling procedure enabled by the microscope software ZEN was used for the acquisition of images larger than the field of view of the microscope objective.

### High-pressure freezing and electron microscopy

Worms were directly frozen without any additives with a high-pressure freezing unit (EMPACT2, Leica), followed by automated freeze substitution (AFS2, Leica) in acetone cocktail (containing 1% osmium tetroxide, 0.1% uranyl acetate, and 0.5% glutaraldehyde), with a slope of 3.0 °C/h, from −90 up to 0 °C (including a rest for 15 h at − 30 °C). At room temperature, the samples were rinsed with acetone and stepwise infiltrated with mixtures of acetone and LX112-resin (Ladd Research) from 1/3 over 1/2 to 2/3 the amount of resin (1.5 h each step). Samples were left in pure resin overnight, then for another 4 h in fresh resin before mounting them between slides and polymerizing at 60 °C. Transverse sections (70 nm) were taken with an ultramicrotome (Ultracut UCT, Leica) and post-contrasted in 1% uranyl acetate in 70% methanol followed by lead citrate. The sections were examined under an electron microscope (Philips Tecnai12, FEI) at 120 kV, and photographs were taken with a TVIPS camera (Tietz).

### Organic extraction and thin-layer chromatography

Frozen worm pellets were homogenized by three rounds of freezing and thawing in an ultrasonication bath and extracted using a standard method [73]. In all experiments, the samples consisted of similar numbers of worms (~ 20,000 larvae). After phase separation, lipids and hydrophilic metabolites were recovered from the organic and aqueous phases, respectively. Non-radioactive samples were normalized for the number of worms. Radioactive ^14^C-acetate-labeled samples were normalized for the number of worms to visualize the rate of catabolism of TGs, phospholipids, trehalose, and amino acids in *daf-2;aak-2* or according to the total radioactivity to determine the state of the gluconeogenesis in different worm strains. In the latter case, the normalization method was chosen to obtain information on the relative abundance of the various metabolites.

TLC was performed on 10 cm HPTLC plates (Merck, Darmstadt, Germany). The running system for sugar detection was chloroform-methanol-water (4:4:1, v/v/v) and chloroform-methanol-water (45:18:3, v/v/v) for phospholipid detection. 2D-TLC for the visualization of hydrophilic metabolites was done using 1-propanol-methanol-ammonia (32%)-water (28:8:7:7, v/v/v/v) as the first system and 1-butanol-acetone-glacial acetic acid-water (35:35:7:23, v/v/v/v) as the second. The TLC plates were sprayed with Molisch reagent for sugar detection, with ninhydrin for visualization of amino acid, and with 3% copper (II) acetate in 10% orthophosphoric acid for imaging of TGs and phospholipids. TLC plates containing radioactive samples were sprayed with EN^3^HANCE spray surface autoradiography enhancer (Perkin Elmer, Waltham, MA, USA) and exposed to X-ray film (Kodak Biomax MR, Sigma-Aldrich, Taufkirchen, Germany). The X-ray films were scanned, and the band intensities of TGs, phosphatidylethanolamines, and trehalose were calculated in Fiji by determining the corresponding optical density peak areas.

### MS Western absolute quantification of metabolic enzymes

Absolute protein quantification was performed using MS Western [48]. All worm strains were washed twice with M9 buffer, counted, collected, and snap-frozen in liquid nitrogen for later analysis. The frozen worms were thawed on ice and crushed using a micro hand mixer (Carl Roth, Germany). The crude extract was then centrifuged for 15 min at 13000 rpm, 4 °C to remove any tissue debris. The clear supernatant was transferred to a fresh Protein Lo-Bind tube (Eppendorf, Hamburg, Germany). The total protein content of the samples was estimated using BCA assay (Thermo Fischer Scientific, Germany), and the equivalent of 60 μg (~ 3500 worms) of total protein content was loaded on to a precast 4 to 20% gradient 1 mm-thick polyacrylamide mini-gels from Anamed Elektrophorese (Rodau, Germany) for 1D SDS-PAGE. Separate gels were run for 1 pmol of BSA and isotopically labeled lysine (K) and arginine (R) incorporated chimeric standard containing 3–5 unique top N quantitypic peptides from 53 metabolic enzymes spanning glycolysis, gluconeogenesis, TCA cycle, and glyoxylate shunt and 5 peptides from the reference protein BSA [48]. Undetectable proteins or proteins without detectable unique sequences like GPD-1, GPD-3, HXK-1, ALH-4, ALH-5, ALH-11, ALH-2, SODH-2, SUCL-1, and SDHD-1 were not included in this analysis. Peptides containing methionine and cysteine were excluded as the former can be variably oxidized and the latter can form disulfide bridges. The sample was cut into six gel slabs, and each slab was co-digested with the bands of BSA and of the chimeric standard using Trypsin Gold, Mass Spectrometry Grade (Promega, Madison). Mass spectra were acquired in data-dependent acquisition mode on a Q-Exactive HF (Thermo Fischer Scientific, Bremen, Germany) mass spectrometer coupled with a Dionex Ultimate 3000 HPLC system (Thermo Scientific, Bremen, Germany). Peptide matching was carried out using Mascot v.2.2.04 software (Matrix Science, London, UK) against *Caenorhabditis elegans* (November 2016) proteome database downloaded from Uniprot. A precursor mass tolerance of 5 ppm and fragment mass tolerance of 0.03 Da were applied: fixed modification, carbamidomethyl (C); variable modifications, acetyl (protein N terminus), oxidation (M); labels: ^13^C_6_-lysine and ^13^C_6_,^15^N_4_-arginine (R); and cleavage specificity, trypsin, with up to 2 missed cleavages allowed. Peptides having the ions score above 15 were accepted (significance threshold *p* < 0.05). The chromatographic alignment and feature detection was carried out using Progenesis LC-MS v.4.1 software (Nonlinear Dynamics, UK). The absolute quantification was performed by calculating the abundances for the labeled and the unlabeled peptide using in-house software.

## Supplementary information

**Additional file 1: Figure S1.** Heat flow of *C. elegans* undergoing reproductive growth or developmental arrest. Left panels - biological replicates generated in various experiments. Right panels - corresponding median heat flow per experiment. Curves representing median values have been smoothed using generalized additive models. **a** Heat flow produced by wild-type (N2) worms undergoing reproductive growth on cholesterol or dauer formation on 4-MS (4-methylated sterol). Corresponding to Fig. 1b. **b** Heat flow of *daf-2* and *daf-7* grown at 25 °C in the presence or absence of DA. **c** Heat flow of *daf-2*, *daf-2;daf-12* and *daf-2;daf-16* grown at 25 °C. Corresponding to Fig. 1d.

**Additional file 2: Figure S2.** Electron micrographs of a *daf-2* arrested L3 larva grown at 25 °C in the presence of DA. The body is not radially constricted but multiple lipid droplets are visible (left panel, arrowheads). Alae are absent (left panel). The gut lumen is elongated with multiple microvilli (central panel, big rectangle on the left panel), and the cuticle has no striated layer (right panel, small rectangle on the left panel). Representative images of five worms. Scale bars 5 μm (left panel), 1 μm (central panel) and 0.5 μm (right panel).

**Additional file 3: Figure S3.** Cumulative heat dissipation of larvae in a developmentally arrested state. Left panels - biological replicates generated in various experiments. Right panels - corresponding median heat per experiment. **a** Heat produced by *daf-2* dauers and *daf-2;daf-12* arrested L3 larvae grown at 25 °C. Corresponding to Fig. 2a. **b** Heat produced by N2 and *daf-16* larvae grown on 4-MS. Corresponding to Fig. 2c.

**Additional file 4: Figure S4.** DAF-16 controls the catabolism of energy reserves in dauer larvae. **a** TLC of lipids and sugars in *daf-2* dauers and *daf-2;daf-12* arrested L3 larvae grown at 25 °C, and wild-type (N2) dauers and *daf-16* dauer-like larvae grown on 4-MS in the period after the developmental arrest is completed. TG – triglycerides, GlcCer – glucosylceramides, Mar – maradolipids, PE – phosphatidylethanolamines, PS – phosphatidylserines, PI – phosphatidylinositols, PC – phosphatidylcholines, Glc – glucose, Tre – trehalose. Representative images of at least 2 experiments. **b** 2D-TLC of amino acids from the same types of animals as in (**a**). Arg – arginine, Lys – lysine, Glu – glutamate, Gly – glycine, Ser - serine, Gln – glutamine, Ala – alanine, Thr - threonine. Representative images of at least 2 experiments.

**Additional file 5: Figure S5.** AAK-2 does not affect exit from dauer state but is required for the preservation of energy reserves in dauers and the gluconeogenic mode in adults. **a** Scheme of the SDS treatment experiment. **b** Survival after SDS treatment. Means + SD of 2 experiments performed in triplicates. **c** 2D-TLC of ^14^C-acetate labeled sugars and amino acids from *daf-2* and *daf-2;aak-2* dauers measured at different time points after the arrest. Tre – trehalose, Glc – glucose, Glu – glutamate, Gly – glycine, Ser - serine, Gln – glutamine, Ala – alanine, Thr - threonine. Representative images from 2 experiments. **d** eGFP localization in *fib-1::eGFP* reproductive and dauer larvae. The outlines of the nuclei are indicated by dashed lines. Maximum intensity Z-projection of the eGFP fluorescence. Scale bars – 5 μm. Representative images of 2 experiments with at least 7 animals. **e** eGFP localization in *daf-2;fib-1::eGFP* and *daf-2;aak-2;fib-1::eGFP* dauers, and *daf-2;fib-1::eGFP* arrested L3 larvae grown on DA at different time points after dauer arrest. Arrow: granules dispersed in the nucleoplasm of some cells. Arrowheads: FIB-1 is almost completely dissolved in the nucleoplasm. Maximum intensity Z-projection of the eGFP fluorescence. Scale bars – 5 μm. Representative images of 3 experiments with at least 7 animals. **f** 2D-TLC of ^14^C-acetate-labeled metabolites from *daf-2* and *daf-2;aak-2* adults grown at 15 °C (left panels) or switched from 15 °C to 25 °C after L4 stage (right panels). Tre – trehalose, Glc – glucose, Glu – glutamate, Gly – glycine, Ser - serine, Gln – glutamine, Ala – alanine, Thr - threonine. Representative images from 2 experiments.

**Additional file 6: Figure S6.** LC-MS/MS (MS Western) analysis of metabolic enzymes. **a**-**f** Multiple peptide-based concordant quantification of an example protein isocitrate lyase (ICL-1). Extracted ion chromatograms (XIC) of ICL-1 endogenous peptides **(a)** LQSAEEAQLWADVFK and **(c)** NQLEGQINLYDAVR, and their corresponding co-eluting labeled peptides **(b** and **d)** from an artificial chimeric standard. **(e)** and **(f)** show the isotopic distribution of the light (L) and heavy (H) peptides. The light-to-heavy ratio (L/H) are similar for both peptides. The quantification is performed by comparing the peak abundances of a known amount of the chimeric standard to the peak abundances of the endogenous peptides to calculate the amount in fmoles. The final amount is reported as an average of the calculated values for all peptides. **g** Scheme of the chimeric construct used in the MS Western measurements. **h** Coefficient of variation (CV %) distribution of 43 proteins were each point represents one protein. The proteome of L3 larvae (*daf-2* at 15 °C) is given as an example. The CV % was calculated for each protein in one sample by the following formula, *σ*(*Quant*^*i*^_*N*_)/*μ*(*Quant*^*i*^_*N*_) where *i* represents the protein, N represents the number of quantitypic peptide in protein (*i)*, and the *Quant* is the amount in fmole. *σ* and *μ* represent the standard deviation and mean, respectively. The median CV was less than 10% (9.336% ± 5.3%). **i** Scatter diagram demonstrating the concordance between peak abundances measured in technical replicates within the whole proteomics data set of L3 and dauer larvae (*daf-2* at 15 °C and 25 °C).

**Additional file 7: Figure S7.** Control of the TCA and glyoxylate cycle. Absolute quantification of enzymes of the TCA cycle and the glyoxylate shunt in *daf-2* and *daf-2;aak-2* grown at 25 °C with or without DA compared to *daf-2* animals at 15 °C. The red arrows and the green circle represent the two competing reactions at the point of divergence between the TCA and the glyoxylate pathway. Means ± standard deviation (S.D.) of 3 biological replicates with 2 technical replicates each.

**Additional file 8: Figure S8.** Control of the gluconeogenesis and the enzyme molar ratios. **a** Absolute quantification of enzymes of the gluconeogenesis and glycolysis in *daf-2* and *daf-2;aak-2* grown at 25 °C with or without DA compared to *daf-2* animals at 15 °C. The red arrows and the green circle represent the two competing reactions at the point of divergence between oxaloacetate recycling and entry into gluconeogenesis. Means ± standard deviation (S.D.) of 3 biological replicates with 2 technical replicates each. **b** Median molar abundance ± S.D. of metabolic pathways. 3 biological replicates with 2 technical replicates each. **c** Median fold change ± S.D. of molar abundances of all proteins in dauers compared to L3 larvae. 3 biological replicates with 2 technical replicates each. **d** Median fold change ± S.D. of molar abundances of proteins according to the metabolic pathways in dauers compared to L3 larvae. 3 biological replicates with 2 technical replicates each. **e** Molar ratio between ICL-1 and IDHG-1. **f** Molar ratio between ICL-1 and IDH-1. **g** Molar ratio between PFK-1 and FBP-1. In **e**, **f**, and **g**: means ± (S.D.) of 3 biological replicates with 2 technical replicates each; *p*-values represent *p* > 0.05 (ns), *p* ≤ 0.05 (*), *p* ≤ 0.01 (**), *p* ≤ 0.001 (***), *p* ≤ 0.0001 (****). One way ANNOVA was performed with Holm-Bonferroni statistical method.

**Additional file 9: Table S1.** Molar abundances of all 43 proteins in biological replicates for all tested conditions.

## Data Availability

The datasets used and/or analyzed during the current study are included in this published article or are available from the corresponding authors on reasonable request.
