# Supplementary figures and images for: A metabolic switch regulates the transition between growth and diapause in C. elegans
Source: BMC Biol. 2020 Mar 18;18:31. doi: 10.1186/s12915-020-0760-3 (PMC7081555; doi:10.1186/s12915-020-0760-3)

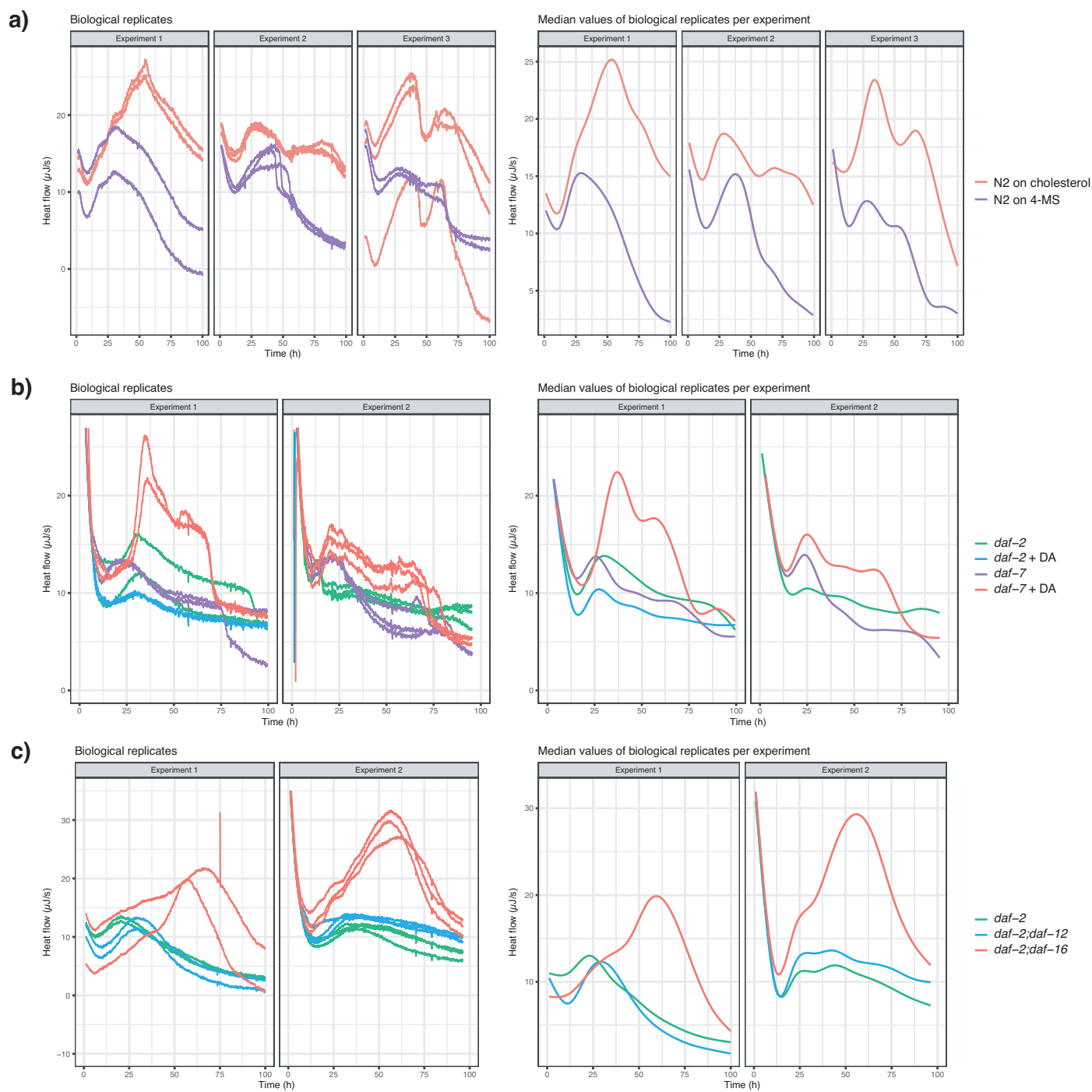

Figure S1  
Penkov et al.

Supplement: Supplementary file 1 — Additional file 1: Figure S1. Heat flow of C. elegans undergoing reproductive growth or developmental arrest. Left panels - biological replicates generated in various experiments. Right panels - corresponding median heat flow per experiment. Curves representing median values have been smoothed using generalized additive models. a Heat flow produced by wild-type (N2) worms undergoing reproductive growth on cholesterol or dauer formation on 4-MS (4-methylated sterol). Corresponding to Fig. 1b. b Heat flow of daf-2 and daf-7 grown at 25 °C in the presence or absence of DA. c Heat flow of daf-2, daf-2;daf-12 and daf-2;daf-16 grown at 25 °C. Corresponding to Fig. 1d. [file 12915_2020_760_MOESM1_ESM.pdf]

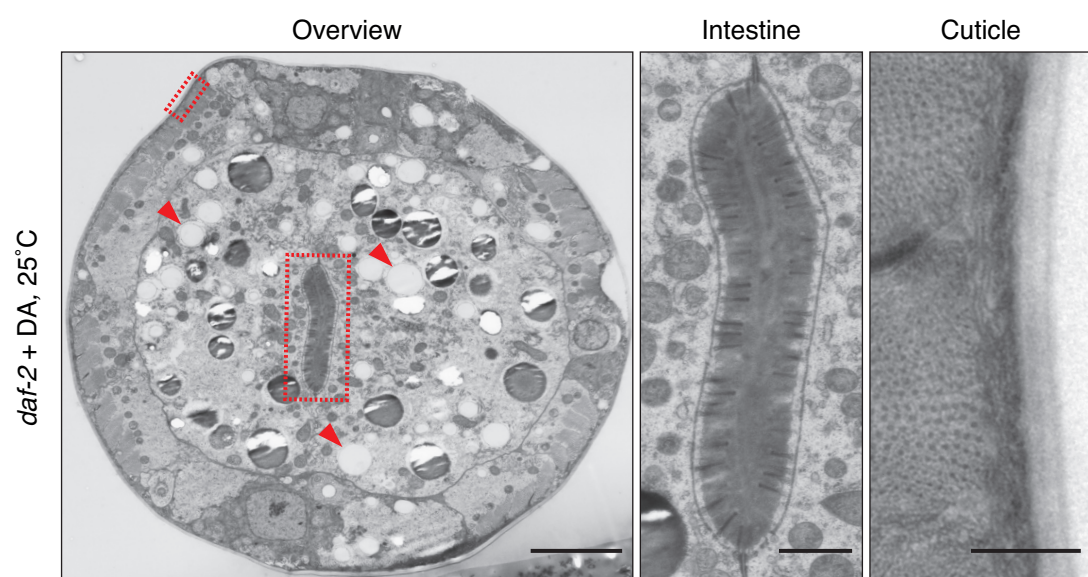

Figure S2  
*Penkov et al.*

Supplement: Supplementary file 2 — Additional file 2: Figure S2. Electron micrographs of a daf-2 arrested L3 larva grown at 25 °C in the presence of DA. The body is not radially constricted but multiple lipid droplets are visible (left panel, arrowheads). Alae are absent (left panel). The gut lumen is elongated with multiple microvilli (central panel, big rectangle on the left panel), and the cuticle has no striated layer (right panel, small rectangle on the left panel). Representative images of five worms. Scale bars 5 μm (left panel), 1 μm (central panel) and 0.5 μm (right panel). [file 12915_2020_760_MOESM2_ESM.pdf]

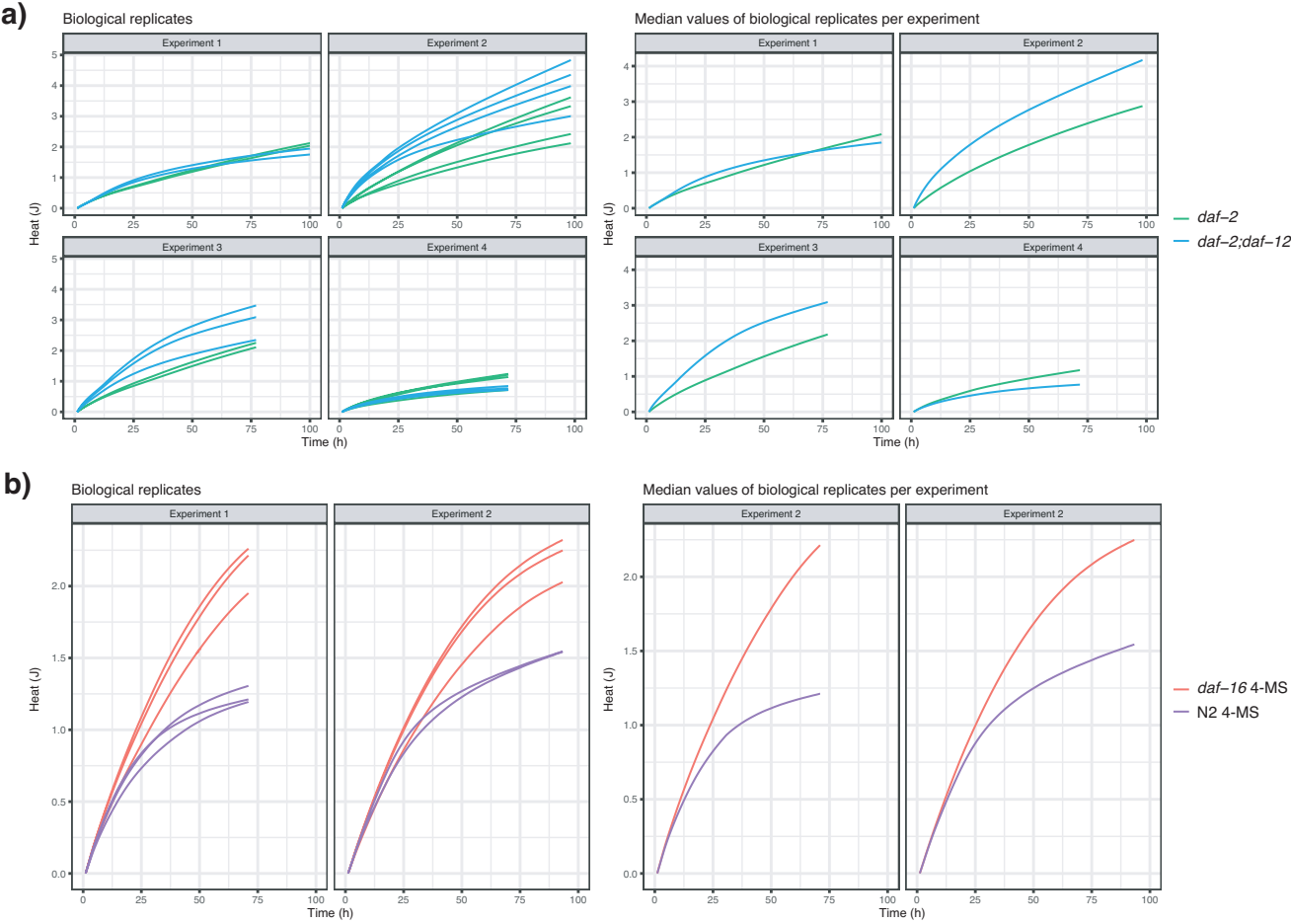

Figure S3  
Penkov et al.

Supplement: Supplementary file 3 — Additional file 3: Figure S3. Cumulative heat dissipation of larvae in a developmentally arrested state. Left panels - biological replicates generated in various experiments. Right panels - corresponding median heat per experiment. a Heat produced by daf-2 dauers and daf-2;daf-12 arrested L3 larvae grown at 25 °C. Corresponding to Fig. 2a. b Heat produced by N2 and daf-16 larvae grown on 4-MS. Corresponding to Fig. 2c. [file 12915_2020_760_MOESM3_ESM.pdf]

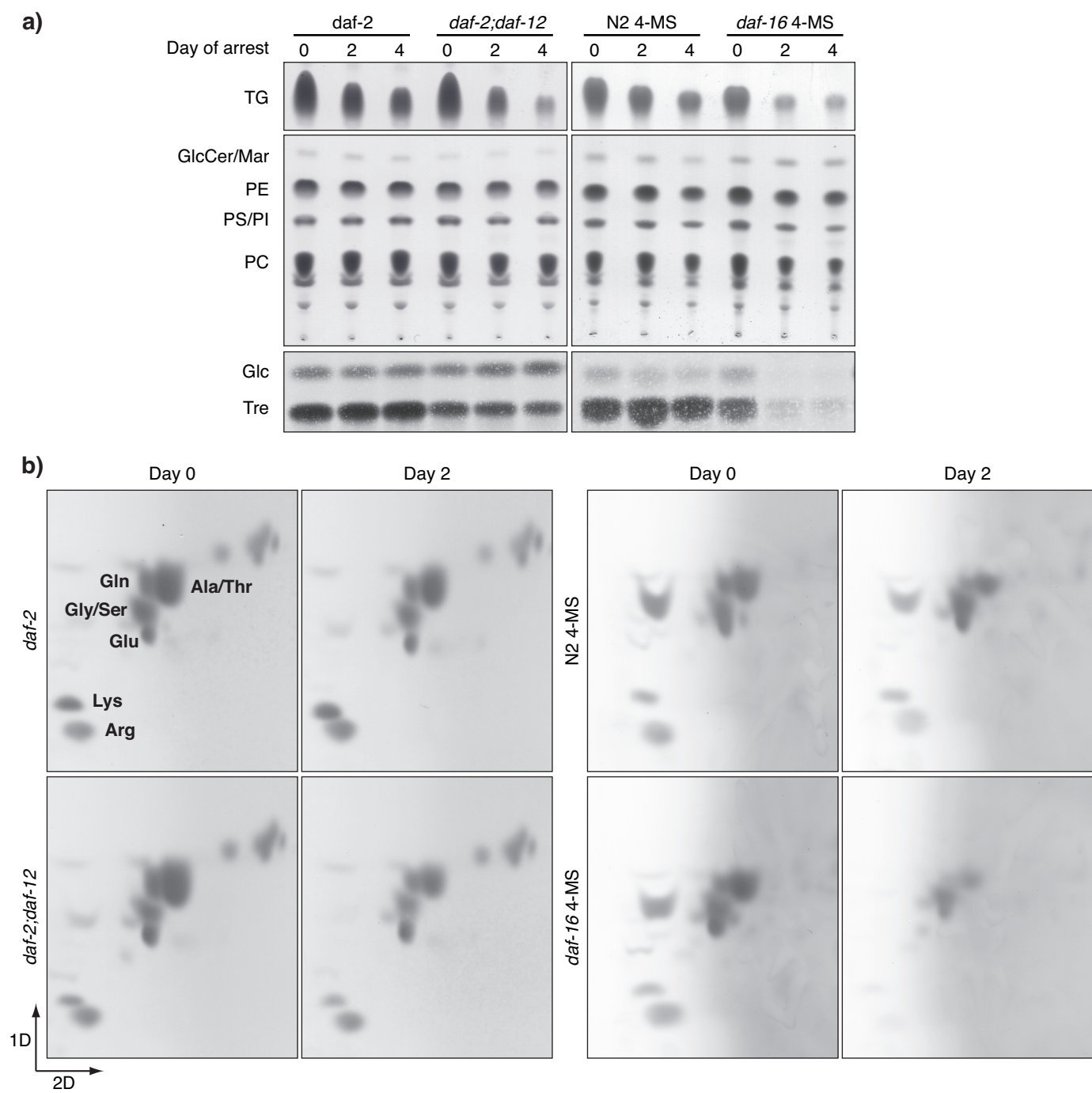

Figure S4  
Penkov et al.

Supplement: Supplementary file 4 — Additional file 4: Figure S4. DAF-16 controls the catabolism of energy reserves in dauer larvae. a TLC of lipids and sugars in daf-2 dauers and daf-2;daf-12 arrested L3 larvae grown at 25 °C, and wild-type (N2) dauers and daf-16 dauer-like larvae grown on 4-MS in the period after the developmental arrest is completed. TG – triglycerides, GlcCer – glucosylceramides, Mar – maradolipids, PE – phosphatidylethanolamines, PS – phosphatidylserines, PI – phosphatidylinositols, PC – phosphatidylcholines, Glc – glucose, Tre – trehalose. Representative images of at least 2 experiments. b 2D-TLC of amino acids from the same types of animals as in (a). Arg – arginine, Lys – lysine, Glu – glutamate, Gly – glycine, Ser - serine, Gln – glutamine, Ala – alanine, Thr - threonine. Representative images of at least 2 experiments. [file 12915_2020_760_MOESM4_ESM.pdf]

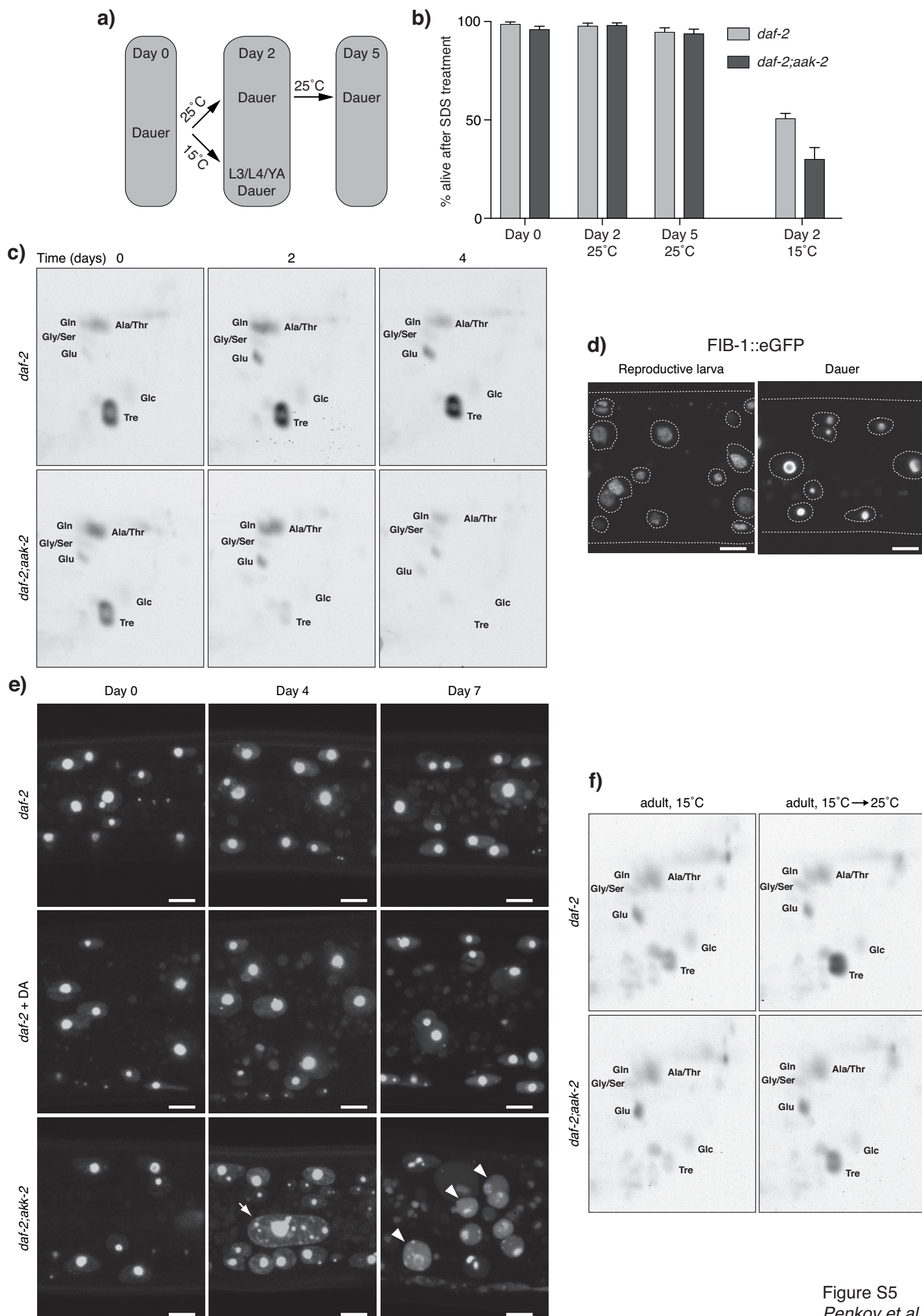

Figure S5  
Penkov et al.

Supplement: Supplementary file 5 — Additional file 5: Figure S5. AAK-2 does not affect exit from dauer state but is required for the preservation of energy reserves in dauers and the gluconeogenic mode in adults. a Scheme of the SDS treatment experiment. b Survival after SDS treatment. Means + SD of 2 experiments performed in triplicates. c 2D-TLC of 14C-acetate labeled sugars and amino acids from daf-2 and daf-2;aak-2 dauers measured at different time points after the arrest. Tre – trehalose, Glc – glucose, Glu – glutamate, Gly – glycine, Ser - serine, Gln – glutamine, Ala – alanine, Thr - threonine. Representative images from 2 experiments. d eGFP localization in fib-1::eGFP reproductive and dauer larvae. The outlines of the nuclei are indicated by dashed lines. Maximum intensity Z-projection of the eGFP fluorescence. Scale bars – 5 μm. Representative images of 2 experiments with at least 7 animals. e eGFP localization in daf-2;fib-1::eGFP and daf-2;aak-2;fib-1::eGFP dauers, and daf-2;fib-1::eGFP arrested L3 larvae grown on DA at different time points after dauer arrest. Arrow: granules dispersed in the nucleoplasm of some cells. Arrowheads: FIB-1 is almost completely dissolved in the nucleoplasm. Maximum intensity Z-projection of the eGFP fluorescence. Scale bars – 5 μm. Representative images of 3 experiments with at least 7 animals. f 2D-TLC of 14C-acetate-labeled metabolites from daf-2 and daf-2;aak-2 adults grown at 15 °C (left panels) or switched from 15 °C to 25 °C after L4 stage (right panels). Tre – trehalose, Glc – glucose, Glu – glutamate, Gly – glycine, Ser - serine, Gln – glutamine, Ala – alanine, Thr - threonine. Representative images from 2 experiments. [file 12915_2020_760_MOESM5_ESM.pdf]

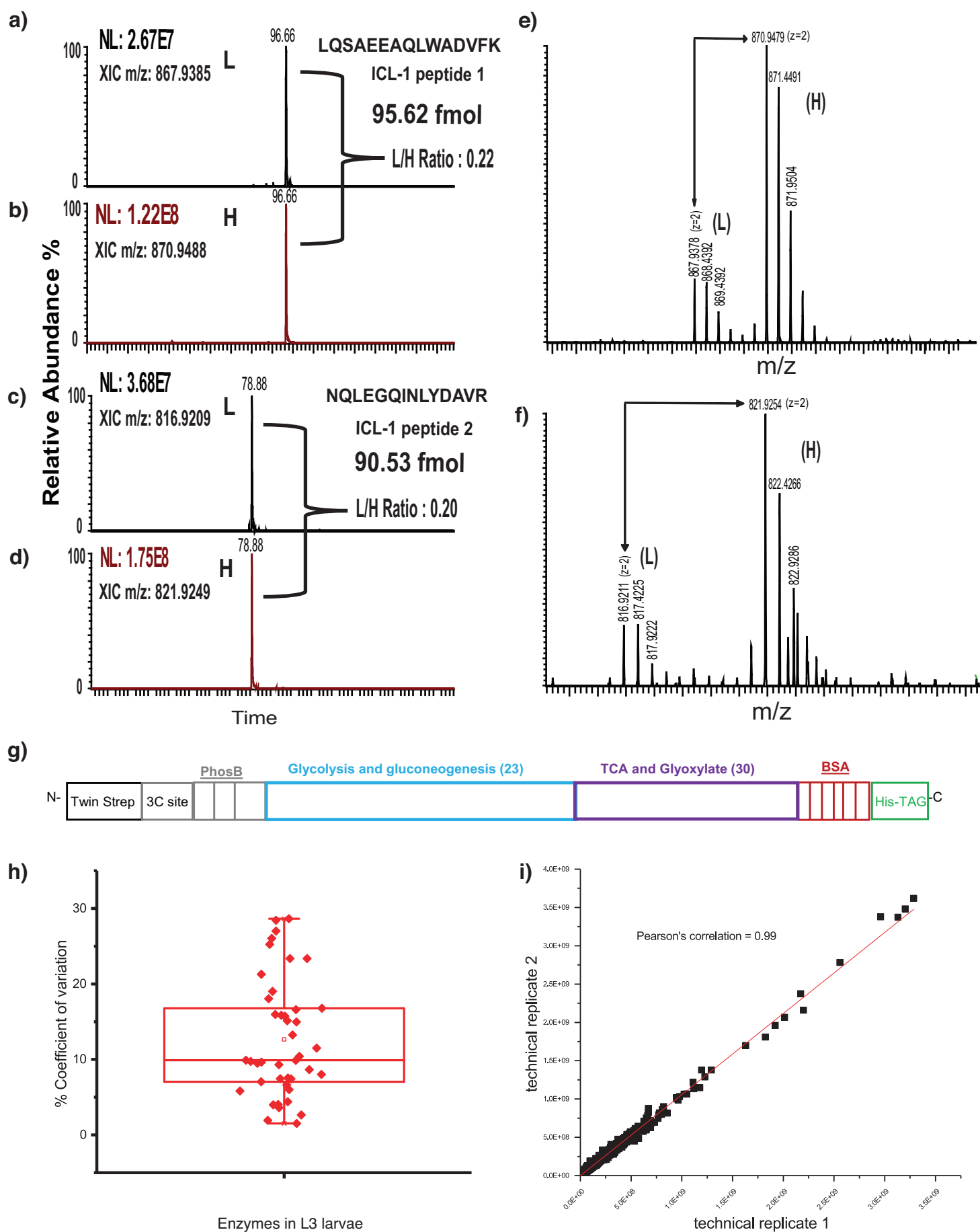

Figure S6  
Penkov et al.

Supplement: Supplementary file 6 — Additional file 6: Figure S6. LC-MS/MS (MS Western) analysis of metabolic enzymes. a-f Multiple peptide-based concordant quantification of an example protein isocitrate lyase (ICL-1). Extracted ion chromatograms (XIC) of ICL-1 endogenous peptides (a) LQSAEEAQLWADVFK and (c) NQLEGQINLYDAVR, and their corresponding co-eluting labeled peptides (b and d) from an artificial chimeric standard. (e) and (f) show the isotopic distribution of the light (L) and heavy (H) peptides. The light-to-heavy ratio (L/H) are similar for both peptides. The quantification is performed by comparing the peak abundances of a known amount of the chimeric standard to the peak abundances of the endogenous peptides to calculate the amount in fmoles. The final amount is reported as an average of the calculated values for all peptides. g Scheme of the chimeric construct used in the MS Western measurements. h Coefficient of variation (CV %) distribution of 43 proteins were each point represents one protein. The proteome of L3 larvae (daf-2 at 15 °C) is given as an example. The CV % was calculated for each protein in one sample by the following formula, σ(QuantiN)/μ(QuantiN) where i represents the protein, N represents the number of quantitypic peptide in protein (i), and the Quant is the amount in fmole. σ and μ represent the standard deviation and mean, respectively. The median CV was less than 10% (9.336% ± 5.3%). i Scatter diagram demonstrating the concordance between peak abundances measured in technical replicates within the whole proteomics data set of L3 and dauer larvae (daf-2 at 15 °C and 25 °C). [file 12915_2020_760_MOESM6_ESM.pdf]

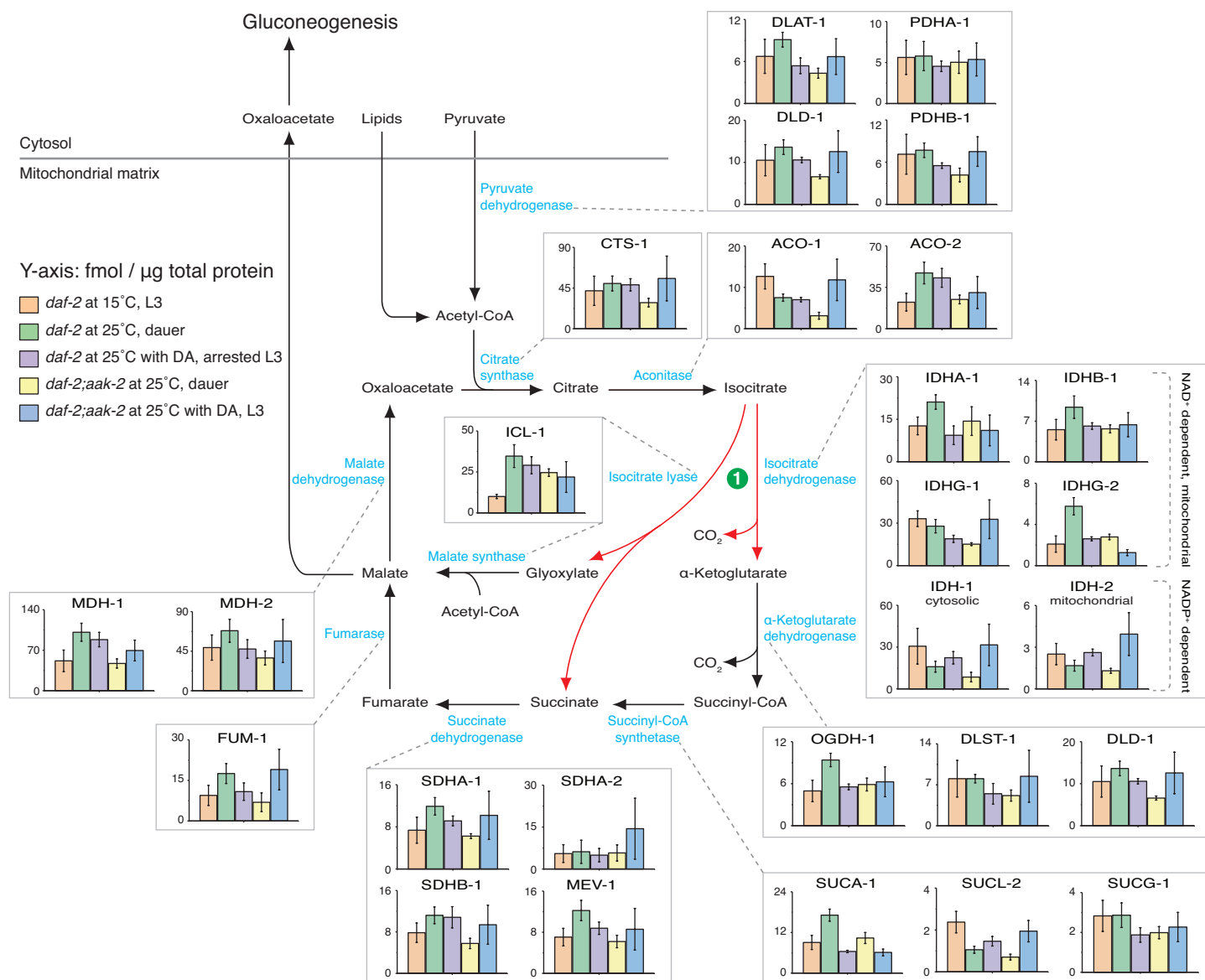

Figure S7  
Penkov et al.

Supplement: Supplementary file 7 — Additional file 7: Figure S7. Control of the TCA and glyoxylate cycle. Absolute quantification of enzymes of the TCA cycle and the glyoxylate shunt in daf-2 and daf-2;aak-2 grown at 25 °C with or without DA compared to daf-2 animals at 15 °C. The red arrows and the green circle represent the two competing reactions at the point of divergence between the TCA and the glyoxylate pathway. Means ± standard deviation (S.D.) of 3 biological replicates with 2 technical replicates each. [file 12915_2020_760_MOESM7_ESM.pdf]

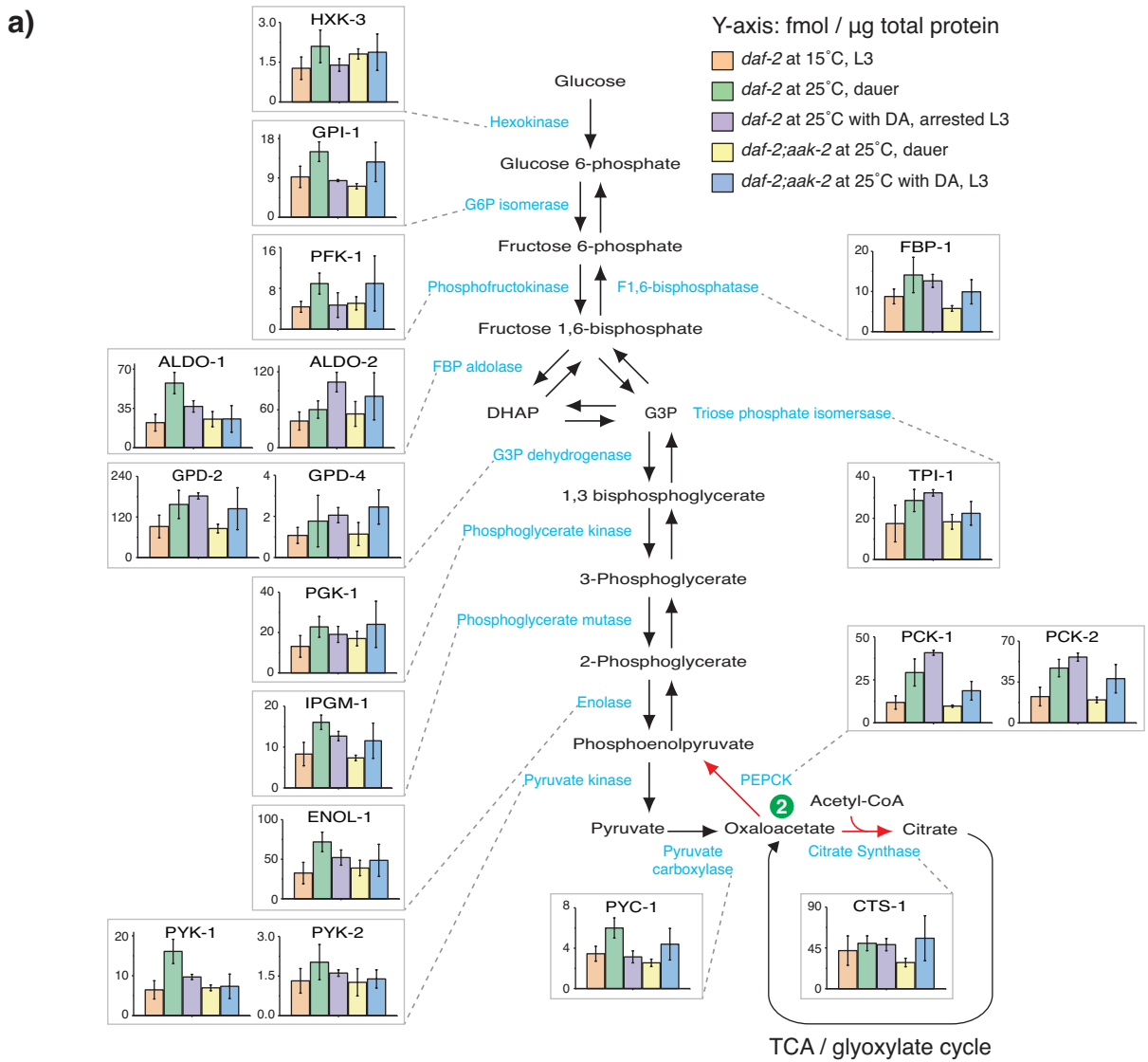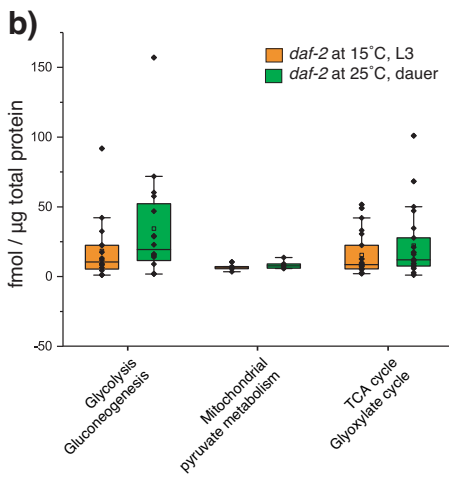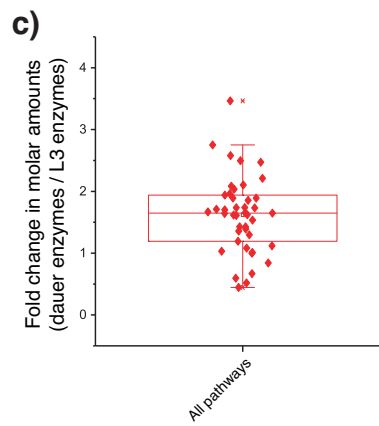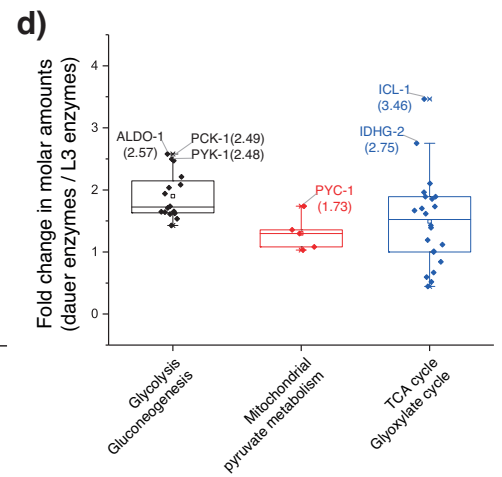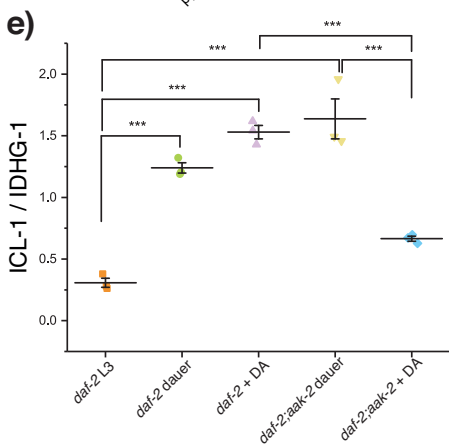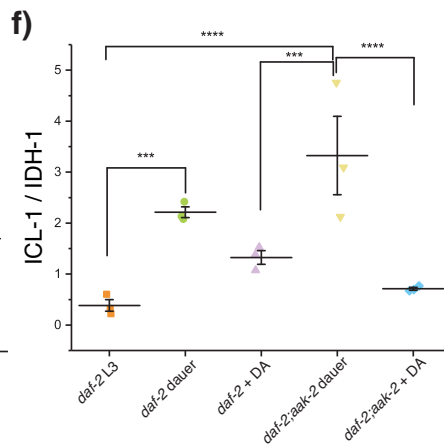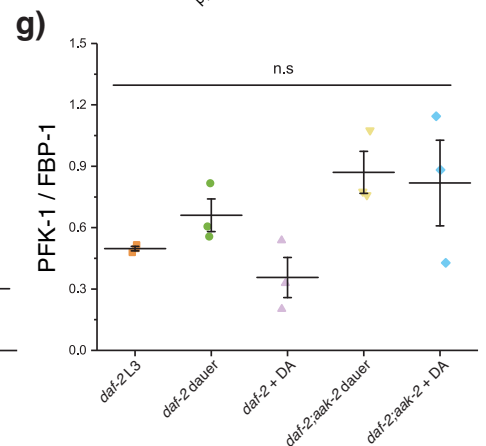

Figure S8  
Penkov et al.

Supplement: Supplementary file 8 — Additional file 8: Figure S8. Control of the gluconeogenesis and the enzyme molar ratios. a Absolute quantification of enzymes of the gluconeogenesis and glycolysis in daf-2 and daf-2;aak-2 grown at 25 °C with or without DA compared to daf-2 animals at 15 °C. The red arrows and the green circle represent the two competing reactions at the point of divergence between oxaloacetate recycling and entry into gluconeogenesis. Means ± standard deviation (S.D.) of 3 biological replicates with 2 technical replicates each. b Median molar abundance ± S.D. of metabolic pathways. 3 biological replicates with 2 technical replicates each. c Median fold change ± S.D. of molar abundances of all proteins in dauers compared to L3 larvae. 3 biological replicates with 2 technical replicates each. d Median fold change ± S.D. of molar abundances of proteins according to the metabolic pathways in dauers compared to L3 larvae. 3 biological replicates with 2 technical replicates each. e Molar ratio between ICL-1 and IDHG-1. f Molar ratio between ICL-1 and IDH-1. g Molar ratio between PFK-1 and FBP-1. In e, f, and g: means ± (S.D.) of 3 biological replicates with 2 technical replicates each; p-values represent p > 0.05 (ns), p ≤ 0.05 (*), p ≤ 0.01 (**), p ≤ 0.001 (***), p ≤ 0.0001 (****). One way ANNOVA was performed with Holm-Bonferroni statistical method. [file 12915_2020_760_MOESM8_ESM.pdf]
